# Changes in Retinal Nerve Fiber Layer Thickness in Patients With Chronic Obstructive Pulmonary Disease: A Systematic Review and Meta‐Analysis

**DOI:** 10.1111/crj.70065

**Published:** 2025-03-04

**Authors:** Yunpeng Xu, Peidong Shi, Xiaoying Liu, Ziyi Jiang, Yanru Chen, Jian Liu, Xunwen Lei, Xue Bai, Fanqi Wu

**Affiliations:** ^1^ The First Clinical Medical College of Lanzhou University Lanzhou China; ^2^ The Second Clinical Medical School of Lanzhou University Lanzhou China; ^3^ Gansu Province Maternity and Child‐Care Hospital (Gansu Provincial Central Hospital) Lanzhou China; ^4^ Department of Ophthalmology The First Hospital of Lanzhou University Lanzhou China; ^5^ Department of Respiratory and Critical Care Medicine The First Hospital of Lanzhou University Lanzhou China; ^6^ Department of Respiratory and Critical Care Medicine The Second Hospital of Lanzhou University Lanzhou China

**Keywords:** chronic obstructive pulmonary disease, meta‐analysis, retinal nerve fiber layer

## Abstract

**Purpose:**

The purpose of this study is to evaluate the relationship between retinal nerve fiber layer (RNFL) thickness and the onset as well as progression of chronic obstructive pulmonary disease (COPD).

**Methods:**

Database searches were conducted in PubMed, Embase, Cochrane Library, Web of Science, CNKI, WanFang Data, VIP Database, and CBM, covering the period from each database's inception to March 2024.

**Results:**

This meta‐analysis included 15 studies from 2016 to 2023, comprising a total of 1455 participants (801 in the COPD group and 654 in the health group). The results showed a significant reduction in RNFL thickness across all quadrants (average, inferior, nasal, superior, and temporal) in the COPD group compared to the health group (MD: −4.46; 95%CI: −7.77 to −1.14; *p* = 0.008; MD: −8.17; 95%CI: −11.36 to −4.99; *p* < 0.00001; MD: −4.69; 95%CI: −7.22 to −2.16; *p* = 0.0003; MD: −4.83; 95%CI: −8.45 to −1.21; *p* = 0.009; MD: −2.89; 95%CI: −5.35 to −0.43; *p* = 0.02). In the mild/moderate COPD group, only the inferior RNFL (MD: −2.32; 95%CI: −4.40 to −0.24; *p* = 0.03) showed a significant reduction. However, in the severe COPD group, all quadrants were significantly reduced (MD: −5.89; 95%CI: −7.40 to −4.38; *p* < 0.0001; MD: −6.74; 95%CI: −10.71 to −2.77; *p* = 0.0009; MD: −4.29; 95%CI: −5.95 to −2.64; *p* < 0.0001; MD: −2.34; 95%CI: −4.30 to −0.37; *p* = 0.02; MD: −4.84; 95%CI: −8.82 to −0.86; *p* = 0.02).

**Conclusion:**

Based on current evidence, the average RNFL thickness and the thicknesses of various RNFL regions in COPD patients are significantly lower than those in healthy subjects, and these reductions are closely associated with disease severity. The inferior RNFL may be the first to show changes with the onset and progression of COPD.

## Graphical abstract

This study evaluates the correlation between the thickness of the retinal nerve fiber layer (RNFL) and the occurrence and progression of chronic obstructive pulmonary disease (COPD). The findings indicate that RNFL thickness in COPD patients is significantly reduced compared to healthy individuals and correlates with disease severity, with the inferior RNFL likely affected earliest.

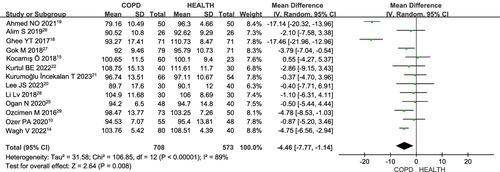

## 1 Introduction

Chronic obstructive pulmonary disease (COPD) is one of the most prevalent respiratory diseases, resulting from long‐term active or passive inhalation of toxic particles or gasses, leading to damage to the small airways, destruction of lung parenchyma, and varying degrees of chronic inflammation [1]. Chronic hypoxia and hypoxia‐related mediators can lead to complications in COPD patients, including weight loss, musculoskeletal dysfunction, cardiovascular diseases, diabetes, and sleep apnea [2, 3, 4]. Chronic hypoxia, along with systemic inflammation, endothelial dysfunction, and increased sympathetic nervous activity, is believed to be associated with changes in ocular structures, including retinal blood vessels and the optic nerve [5, 6, 7]. Therefore, ocular biomarkers associated with COPD have become an emerging research focus in recent years. Changes in ocular hemodynamics associated with COPD may impair blood perfusion to the optic nerve head, reducing the oxygen and nutrients available to the RNFL. This insufficiency results in ganglion cell loss, which subsequently causes RNFL cell damage or death, reduced RNFL thickness, and an increased risk of ocular complications such as glaucoma [8, 9, 10].

The measurement of RNFL thickness is an important reference for the early detection of glaucoma [11]. Glaucoma, according to the World Health Organization, is the second leading cause of blindness worldwide, with an unclear etiology that results in irreversible vision loss. As ganglion cell axons progress, vision impairment gradually becomes apparent [12]. Early measurement of RNFL thickness is critical for diagnosing and preventing eye complications such as glaucoma and optic nerve atrophy [12, 13]. Small‐sample studies have found that as the severity of COPD increases, RNFL thickness decreases. Other studies have reported that, compared to healthy control groups, the average RNFL thickness during exacerbations and stable periods of COPD patients is similar [14, 15]. Currently, no evidence‐based research exists on the link between COPD occurrence, its severity, and changes in RNFL. Therefore, this study employs meta‐analysis to assess the connection between RNFL and COPD incidence and severity, exploring the potential association between COPD and optic nerve pathology.

## 2 Material and Methods

The study adheres to PRISMA guidelines and is based on a protocol registered in the PROSPERO systematic review database (#CRD42024516479).

### 2.1 Search Strategy

Searches were conducted in multiple databases including PubMed, Embase, Cochrane Library, Web of Science, CNKI, WanFang Data, VIP, and CBM, covering the period from each database's inception to March 2024. Both MeSH and free‐text terms were used. Detailed search terms and strategies for this meta‐analysis are provided in the Supporting Information.

### 2.2 Eligibility Criteria

The included studies must involve COPD patients diagnosed according to the standards set by the American Thoracic Society or the Global Initiative for Chronic Obstructive Lung Disease (GOLD). Based on GOLD standards, patients are categorized into four groups according to airflow limitation, exercise capacity (modified Medical Research Council [mMRC] score), and COPD Assessment Test (CAT) score: Group A (mild) includes patients with 50% ≤ Forced Expiratory Volume in 1 s (FEV_1_) < 80%, CAT < 10, mMRC = 0–1, and one or no hospitalizations due to acute exacerbation; Group B (moderate) with 50% ≤ FEV_1_ < 80%, CAT ≥ 10, mMRC ≥ 2, and one or no hospitalizations due to acute exacerbation; Group C (severe) with FEV_1_ < 50%, CAT < 10, mMRC = 0–1, and a history of two acute exacerbations or one hospitalization; and Group D (very severe) with FEV_1_ < 50%, CAT ≥ 10, mMRC ≥ 2, and a history of two acute exacerbations or one hospitalization. We divide COPD patients into two subgroups according to this classification standard: mild/moderate COPD (including Groups A and B) and severe COPD (including Groups C and D); describe the relationship between RNFL and COPD; and report on the RNFL thickness (in μm) levels of COPD patients and healthy control groups; included patients have undergone ophthalmic system examination, without glaucoma, hypertensive retinopathy, diabetic retinopathy, ocular trauma, history of eye surgery, and so on.

### 2.3 Exclusion Criteria

Studies to be excluded must meet the following criteria: reviews, conference papers, letters, and studies published repetitively; studies without a control group; studies that do not report the outcome indicators required for this research; and studies and animal experiments that involve conditions other than COPD diagnosis.

### 2.4 Selection and Data Extraction

Two researchers independently screened the literature, extracted data, and cross‐checked the results, with any disagreements resolved through discussion or third‐party arbitration. Titles and abstracts were initially screened, followed by full‐text assessments to determine the final inclusion of studies. The extracted data included the first author, year of publication, sample size, study design, average age, body mass index (BMI), intraocular pressure (IOP), axial length of the eye, FEV_1_ (%), FEV_1_ (L), forced expiratory volume in 1 s to forced vital capacity ratio (FEV_1_/FVC) (%), disease duration, RNFL thickness for each group, and risk of bias assessment criteria.

### 2.5 Quality Assessment

All studies were assessed using a specialized tool by the National Institutes of Health (NIH), where each study was rated as poor, fair, or good [16]. This approach ensures a comprehensive and objective assessment of the studies included in the meta‐analysis.

### 2.6 Statistical Analysis

Meta‐analyses were conducted using RevMan 5.4 and Stata 15.1 software. The studies included continuous variables, with mean difference (*MD*) and 95% confidence intervals (*CI*) serving as the effect measures. Heterogeneity was assessed using the *Q* test and *I* ^2^ values. A fixed‐effect model was applied when *p* > 0.1 or *I* ^2^ < 50%, indicating no significant heterogeneity; otherwise, a random‐effects model was applied. For outcomes with substantial heterogeneity, subgroup or sensitivity analyses were conducted to identify its sources. When more than 10 studies were included for an outcome, Egger's test was used to assess publication bias [17].

## 3 Results

**FIGURE 1:**
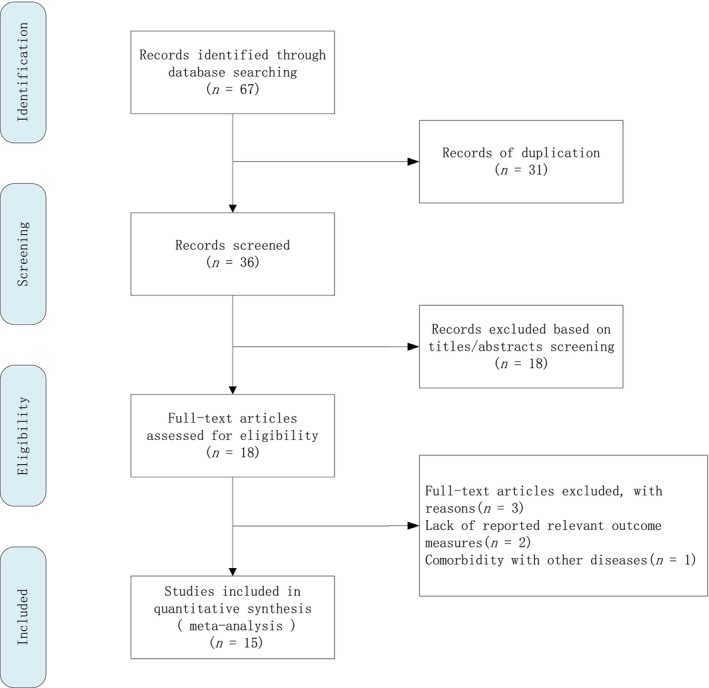
Flow chart of literature screening.

In the preliminary search, a total of 67 articles were identified, including 13 Chinese and 54 English articles. After deduplication, 36 articles remained. Titles and abstracts were screened to exclude irrelevant articles, and 18 full texts were obtained for further review. After full‐text review, three articles were excluded: two for not reporting relevant outcome indicators and one for including participants with other diseases. Ultimately, 15 articles [8, 14, 15, 18, 19, 20, 21, 22, 23, 24, 25, 26, 27, 28, 29] were included in the study, comprising 1 Chinese and 14 English articles, published between 2016 and 2023. The total sample size was 1455, with 801 participants in the COPD group and 654 in the health group. The literature screening process is illustrated in (Figure 1). The overall quality of the included studies was considered high. The characteristics of the included studies are shown in Table S1. The results of the bias risk assessment for observational cohort studies and cross‐sectional studies included in the literature are shown in Table S2. The results of the bias risk assessment for case–control studies included in the literature are shown in Table S3.

### 3.1 Average RNFL in COPD Versus Health

**FIGURE 2:**
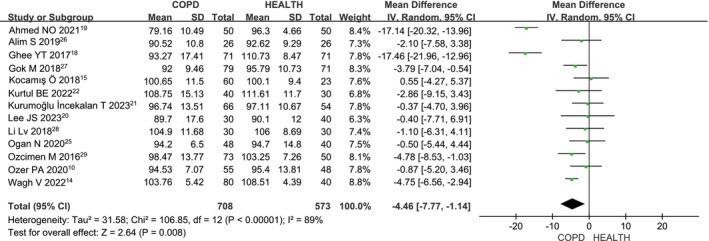
Forest plot of the average RNFL level between COPD and health patients.

Thirteen studies [14, 15, 18, 19, 20, 21, 22, 24, 25, 26, 27, 28, 29] assessed the average RNFL thickness in COPD patients compared to healthy individuals. Due to significant heterogeneity among the studies (*I* ^2^ = 89%, *p* < 0.00001), a random‐effects model was used. The meta‐analysis (MD: −4.46; 95% CI: −7.77 to −1.14; *p* = 0.008; Figure 2) shows that COPD patients have a significantly lower average RNFL thickness than the healthy control group, with statistically significant results.

### 3.2 Inferior RNFL in COPD Versus Health

**FIGURE 3:**
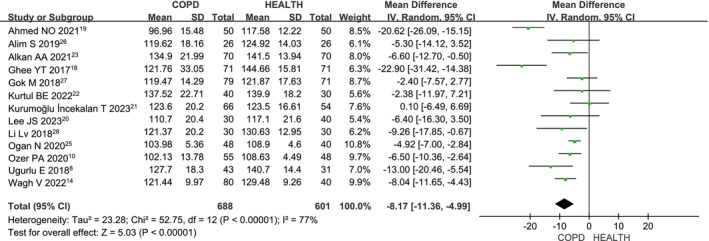
Forest plot of the inferior RNFL level between COPD and health patients.

Thirteen studies [8, 14, 18, 19, 20, 21, 22, 23, 24, 25, 26, 27, 28] investigated the inferior RNFL thickness in COPD patients compared to healthy individuals. Given the heterogeneity among the studies (*I* ^2^ = 77%, *p* < 0.00001), a random‐effects model was applied. The meta‐analysis (MD: −8.17; 95% CI: −11.36 to −4.99; *p* < 0.00001; Figure 3) shows a significantly lower inferior RNFL thickness in COPD patients than in healthy controls, with statistically significant results.

### 3.3 Nasal RNFL in COPD Versus Health

**FIGURE 4:**
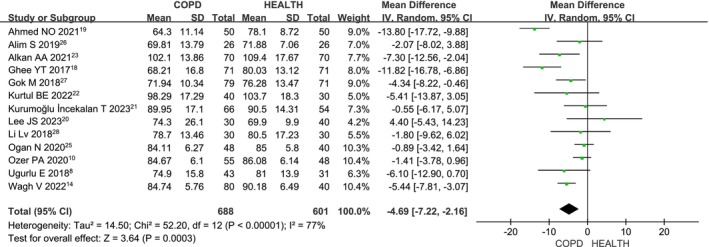
Forest plot of the nasal RNFL level between COPD and health patients.

Thirteen studies [8, 14, 18, 19, 20, 21, 22, 23, 24, 25, 26, 27, 28] assessed nasal RNFL thickness in COPD patients compared to healthy individuals. Due to the heterogeneity among the studies (*I* ^2^ = 77%, *p* < 0.00001), a random‐effects model was used. The meta‐analysis (MD: −4.69; 95% CI: −7.22 to −2.16; *p* = 0.0003; Figure 4) shows that nasal RNFL thickness is significantly reduced in COPD patients compared to healthy controls, with statistically significant findings.

### 3.4 Superior RNFL in COPD Versus Health

**FIGURE 5:**
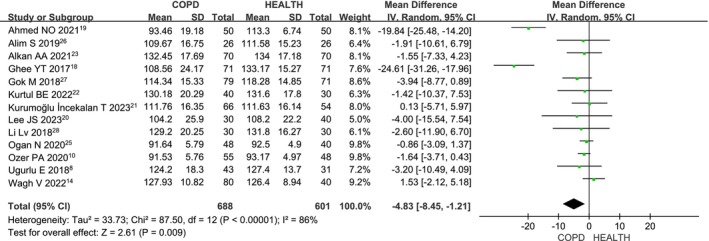
Forest plot of the superior RNFL level between COPD and health patients.

Thirteen studies [8, 14, 18, 19, 20, 21, 22, 23, 24, 25, 26, 27, 28] evaluated superior RNFL thickness in COPD patients versus healthy controls. Given the heterogeneity (*I* ^2^ = 86%, *p* < 0.00001), a random‐effects model was applied. The meta‐analysis (MD: −4.83; 95% CI: −8.45 to −1.21; *p* = 0.009; Figure 5) shows that superior RNFL thickness is significantly reduced in COPD patients compared to healthy controls, with statistically significant outcomes.

### 3.5 Temporal RNFL in COPD Versus Health

**FIGURE 6:**
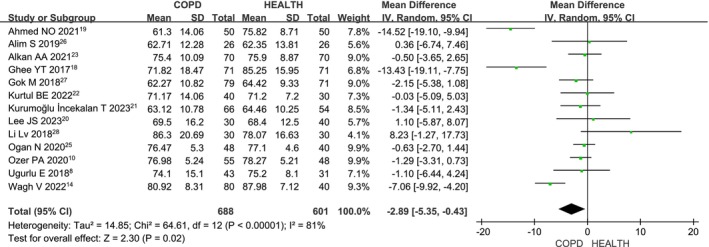
Forest plot of the temporal RNFL level between COPD and health patients.

Thirteen studies [8, 14, 18, 19, 20, 21, 22, 23, 24, 25, 26, 27, 28] assessed temporal RNFL thickness in COPD patients versus healthy controls. Due to heterogeneity (*I* ^2^ = 81%, *p* < 0.00001), a random‐effects model was applied. The meta‐analysis (MD: −2.89; 95% CI: −5.35 to −0.43; *p* = 0.02; Figure 6) shows that temporal RNFL thickness is significantly reduced in COPD patients compared to healthy controls, with statistically significant results.

### 3.6 RNFL in Mild/Moderate COPD Versus Health

Six studies [14, 15, 21, 22, 25, 27] examined average RNFL thickness in mild/moderate COPD patients versus healthy controls. With minimal heterogeneity (*I* ^2^ = 8%, *p* = 0.37), a fixed‐effect model was used. The meta‐analysis (MD: −0.91; 95% CI: −2.39 to 0.57; *p* = 0.23; Table 1) found no statistically significant difference between the two groups.

**TABLE 1:** Comparison of RNFL thickness in different quadrants between COPD patients with different severity levels and healthy controls.

Outcomes	Number of studies	Heterogeneity test results		Effects model	Meta‐analysis results	
		*p* value	*I* ^2^ value (%)		Pooled effect size MD (95% CI)	*p* value
Mild/moderate COPD vs. health						
Average RNFL	6	0.37	8	Fixed‐effect model	−0.91 (−2.39, 0.57)	0.23
Inferior RNFL	5	0.51	0	Fixed‐effect model	−2.32 (−4.40, −0.24)	0.03
Nasal RNFL	5	0.03	64	Random‐effects model	−1.89 (−5.34, 1.56)	0.28
Superior RNFL	5	0.33	13	Fixed‐effect model	1.77 (−0.26, 3.81)	0.09
Temporal RNFL	5	0.02	67	Random‐effects model	−0.16 (−3.06, 2.75)	0.92
Severe COPD vs. health						
Average RNFL	6	0.08	49	Fixed‐effect model	−5.89 (−7.40, −4.38)	*p* < 0.00001
Inferior RNFL	5	0.03	64	Random‐effects model	−6.74 (−10.71, −2.77)	0.0009
Nasal RNFL	5	0.27	23	Fixed‐effect model	−4.29 (−5.95, −2.64)	*p* < 0.00001
Superior RNFL	5	0.74	0	Fixed‐effect model	−2.34 (−4.30, −0.37)	0.02
Temporal RNFL	5	0.0001	83	Random‐effects model	−4.84 (−8.82, −0.86)	0.02

Five studies [14, 21, 22, 25, 27] compared RNFL thickness in different quadrants between mild/moderate COPD patients and healthy controls. For the inferior RNFL, no heterogeneity was observed (*I* ^2^ = 0%, *p* = 0.51), so a fixed‐effect model was applied. The analysis (MD: −2.32; 95% CI: −4.40 to 0.24; *p* = 0.03; Table 1) showed a significantly lower inferior RNFL thickness in mild/moderate COPD patients. The nasal RNFL showed heterogeneity (*I* ^2^ = 64%, *p* = 0.03), requiring a random‐effects model, but the results (MD: −1.89; 95% CI: −5.34 to 1.56; *p* = 0.28; Table 1) showed no significant difference. For the superior RNFL, minimal heterogeneity (*I* ^2^ = 13%, *p* = 0.33) permitted the use of a fixed‐effect model, with the results (MD: 1.77; 95% CI: −0.26 to 3.81; *p* = 0.09; Table 1) showing no significant difference. Significant heterogeneity in the temporal RNFL (*I* ^2^ = 67%, *p* = 0.02) led to the use of a random‐effects model, and the analysis (MD: −0.16; 95% CI: −3.06 to 2.75; *p* = 0.92; Table 1) also showed no significant difference between the groups.

Thus, the inferior RNFL thickness in mild/moderate COPD patients was significantly thinner than in healthy controls, with statistical significance. No statistically significant differences were found in other quadrants.

### 3.7 RNFL in Severe COPD Versus Health

Six studies [14, 15, 21, 22, 25, 27] evaluated average RNFL thickness in severe COPD patients versus healthy individuals. Given moderate heterogeneity (*I* ^2^ = 49%, *p* = 0.08), a fixed‐effect model was used. The meta‐analysis (MD: −5.89; 95% CI: −7.40 to −4.38; *p* < 0.00001; Table 1) shows a significantly lower average RNFL thickness in severe COPD patients compared to healthy controls.

Five studies [14, 21, 22, 25, 27] examined RNFL thickness in different quadrants between severe COPD patients and healthy individuals. For the inferior RNFL, heterogeneity (*I* ^2^ = 64%, *p* = 0.03) required a random‐effects model, revealing (MD: −6.74; 95% CI: −10.71 to −2.77; *p* = 0.0009; Table 1) significantly lower thickness in severe COPD patients. The nasal RNFL had minimal heterogeneity (*I* ^2^ = 23%, *p* = 0.27), allowing a fixed‐effect model, with results (MD: −4.29; 95% CI: −5.95 to −2.64; *p* < 0.00001; Table 1) indicating significantly lower thickness in severe COPD patients. The superior RNFL showed no heterogeneity (*I* ^2^ = 0%, *p* = 0.74), and the fixed‐effect model revealed (MD: −2.34; 95% CI: −4.30 to −0.37; *p* = 0.02; Table 1) significantly reduced thickness in severe COPD patients. Significant heterogeneity in the temporal RNFL (*I* ^2^ = 83%, *p* = 0.0001) necessitated a random‐effects model, showing (MD: −4.84; 95% CI: −8.82 to −0.86; *p* = 0.02; Table 1) significantly lower thickness in severe COPD patients compared to healthy controls.

Thus, in severe COPD patients, the RNFL thickness in different quadrants is markedly reduced compared to healthy controls, with statistically significant findings.

### 3.8 RNFL in Mild/Moderate COPD Versus Severe COPD

Six studies [14, 15, 21, 22, 25, 27] compared average RNFL thickness between mild/moderate and severe COPD patients. With no heterogeneity (*I* ^2^ = 0%, *p* = 0.65), a fixed‐effect model was applied. The meta‐analysis (MD: 5.06; 95% CI: 3.58–6.55; *p* < 0.00001; Table 2) shows significantly lower average RNFL thickness in severe COPD patients compared to those with mild/moderate COPD.

**TABLE 2:** Comparison of RNFL thickness in different quadrants between Mild/Moderate COPD and severe COPD patients.

Outcomes	Number of studies	Heterogeneity test results		Effects model	Meta‐analysis results	
		*p* value	*I* ^2^ value (%)		Pooled effect size MD (95% CI)	*p* value
Average RNFL	6	0.65	0	Fixed‐effect model	5.06 (3.58, 6.55)	*p* < 0.00001
Inferior RNFL	5	0.13	44	Fixed‐effect model	4.84 (2.63, 7.04)	*p* < 0.0001
Nasal RNFL	5	0.53	0	Fixed‐effect model	2.08 (0.26, 3.90)	0.02
Superior RNFL	5	0.47	0	Fixed‐effect model	4.13 (1.83, 6.43)	0.0004
Temporal RNFL	5	0.04	59	Random‐effects model	4.94 (2.02, 7.87)	0.0009

Five studies [14, 21, 22, 25, 27] evaluated RNFL thickness in different quadrants between these groups. For the inferior RNFL, minimal heterogeneity (*I* ^2^ = 44%, *p* = 0.13) allowed a fixed‐effect model, revealing (MD: 4.84; 95% CI: 2.63–7.04; *p* < 0.0001; Table 2) significantly lower thickness in severe COPD patients. The nasal RNFL showed no heterogeneity (*I* ^2^ = 0%, *p* = 0.53), so a fixed‐effect model was used, with results (MD: 2.08; 95% CI: 0.26–3.90; *p* = 0.02; Table 2) indicating significantly lower thickness in severe COPD patients. For the superior RNFL, the absence of heterogeneity (*I* ^2^ = 0%, *p* = 0.47) led to a fixed‐effect model, showing (MD: 4.13; 95% CI: 1.83–6.43; *p* = 0.0004; Table 2) significantly lower thickness in severe COPD patients. The temporal RNFL displayed significant heterogeneity (*I* ^2^ = 59%, *p* = 0.04), requiring a random‐effects model, and the analysis (MD: 4.94; 95% CI: 2.02–7.87; *p* = 0.0009; Table 2) confirmed significantly lower thickness in severe COPD patients.

Thus, the RNFL thickness in different quadrants is significantly lower in severe COPD patients compared to those with mild/moderate COPD, with statistically significant findings.

### 3.9 Sensitivity Analysis

Sensitivity analyses were conducted for outcome indicators with high heterogeneity by sequentially excluding each study. It was found that in the “RNFL in mild/moderate COPD versus health” for the nasal RNFL outcome, excluding the study by Wagh et al. [14] changed *I* ^2^ from 64% to 21%. A reanalysis of the meta‐analysis showed MD: 0.13; 95% CI: −2.31 to 2.57; *p* = 0.92, indicating no significant change in the results. For the temporal RNFL outcome in the same comparison, excluding the study by Wagh et al. [14] changed *I* ^2^ from 67% to 47%. The reanalysis showed MD: 1.58; 95% CI: −0.07 to 3.24; *p* = 0.06, indicating no significant change in the results. In the “RNFL in severe COPD versus health” for the inferior RNFL outcome, excluding the study by Wagh et al. [14] changed *I* ^2^ from 64% to 0%. The reanalysis showed MD: −5.40; 95% CI: −7.46 to −3.34; *p* < 0.00001, indicating no significant change in the results. For the temporal RNFL outcome in the same comparison, excluding the study by Wagh et al. [14] changed *I* ^2^ from 83% to 0%. The reanalysis showed (MD: −2.69; 95% CI: −4.44 to −0.95; *p* = 0.002), indicating no significant change in the results. In the “RNFL in mild/moderate COPD versus severe COPD” for the temporal RNFL outcome, excluding the study by Wagh et al. [14] changed *I* ^2^ from 59% to 46%. The reanalysis showed MD: 4.21; 95% CI: 2.30 to 6.11; *p* < 0.00001, indicating no significant change in the results.

In summary, the study by Wagh et al. [14]. might be a source of heterogeneity in this meta‐analysis. However, excluding this study found the results to remain stable, suggesting that the heterogeneity observed is acceptable.

### 3.10 Publication Bias Analysis

Because the number of studies included in the outcome indicators for RNFL thickness comparison between COPD and HEALTH exceeds 10, Egger's test was conducted using Stata 15.1 software. The *p* values for average RNFL, inferior RNFL, nasal RNFL, superior RNFL, and temporal RNFL were 0.957, 0.318, 0.598, 0.953, and 0.348, respectively. Therefore, no publication bias was detected in any of these outcomes. Egger's publication bias plot is shown in Figures S16–S20.

## 4 Discussion

This meta‐analysis investigates the potential association between RNFL thickness and the occurrence and progression of COPD. A total of 15 studies [8, 14, 15, 18, 19, 20, 21, 22, 23, 24, 25, 26, 27, 28, 29] were included. The results showed that COPD patients had significantly lower average RNFL thickness compared to healthy controls, consistently observed across the inferior, nasal, superior, and temporal quadrants. Subsequently, COPD patients were stratified into mild/moderate and severe groups according to disease severity. It was found that, compared to healthy individuals, only the RNFL thickness in the inferior quadrant was significantly lower in mild/moderate COPD patients, with no similar findings in other quadrants. In contrast, when severe COPD patients were compared to healthy individuals, the RNFL thickness was significantly reduced in the severe COPD group, both on average and across different quadrants. Lastly, a comparison between mild/moderate and severe COPD patients revealed that the average RNFL and the RNFL thickness in each quadrant were significantly lower in the severe COPD group. These findings suggest that RNFL thickness progressively decreases as COPD advances, with the inferior RNFL quadrant being the first to show significant changes.

RNFL thickness is a key indicator in the quantitative assessment of optic nerve damage [30]. Studies involving 8941 subjects have found that suffering from COPD increases the risk of concurrent glaucoma in women, with the risk being more pronounced in nonsmoking women [31]. Inhaled corticosteroids are a standard treatment for COPD, significantly reducing mortality, improving lung function, and decreasing the frequency of acute exacerbations, especially in patients with eosinophilic COPD [32, 33]. Although the incidence of adverse events with inhaled corticosteroids is much lower than with systemic corticosteroids, Gartlehner et al. [32] conducted a meta‐analysis of 13 RCT studies and found that inhaled corticosteroids in treating COPD patients are still associated with cataracts and open‐angle glaucoma. However, the mechanism through which COPD may lead to eye diseases as the disease progresses is still unclear. Research has confirmed that most glaucoma patients exhibit anatomical abnormalities before eye symptoms appear, and by the time significant eye symptoms occur, RNFL damage has already reached 20%–40% [11]. Therefore, COPD patients may have a higher likelihood of experiencing visual field defects compared to the general population. Consequently, early detection of RNFL thickness in COPD patients may be clinically significant for preventing ocular complications.

In COPD patients, the significant reduction in RNFL thickness may be associated with decreased retinal microvascular density. The central retinal and posterior ciliary arteries are essential for supplying blood to the peripapillary capillary plexus in a radial pattern around the optic disk. Studies employing color Doppler ultrasound have found that in COPD patients, increased resistance in the central retinal and posterior ciliary arteries reduces blood flow to the peripapillary capillary plexus [9, 10]. This results in the RNFL being in a state of long‐term hypoxia and nutrient deficiency, leading to direct neuronal damage [9, 10]. Chronic hypoxia, inflammatory factors, oxidative stress, and smoking are considered causes of endothelial dysfunction in COPD patients [34, 35]. In healthy individuals, the balance between vasoconstrictors like endothelin‐1 (ET‐1) and vasodilators like nitric oxide (NO) dynamically regulates the tension of ocular microvessels, which primarily determines ocular blood flow pressure and distribution [36, 37]. In COPD patients, elevated ET‐1 levels in sputum, plasma, and urine may disrupt the regulation of ocular microvessels, leading to abnormal blood flow distribution and changes in RNFL thickness [38, 39].

Existing meta‐analyses have found that patients with obstructive sleep apnea (OSA) have significantly reduced average RNFL thickness throughout the week compared to the normal population [40]. This thinning becomes more pronounced with the severity of the disease, likely due to the repeated apnea or hypoventilation during sleep in OSA patients. COPD patients, on the other hand, exhibit an adaptive protective mechanism in which cerebral blood flow increases compensatorily when arterial oxygen saturation decreases, ensuring adequate oxygen supply to the brain [41]. However, half of the COPD patients lose this mechanism during nonrapid eye movement (NREM) sleep, leading to uncompensated hypoxemia during these periods, which results in cerebral hypoxia and potential neural damage, thus affecting the RNFL [41]. Smoking is a major risk factor for COPD and a leading cause of endothelial dysfunction and microvascular abnormalities. Nicotine activates the sympathetic nervous system, causing vasoconstriction [42]. Studies have shown that compared to nonsmokers, individuals who smoke more than 20 cigarettes a day exhibit a significant increase in retinal vein diameter, which can be reversed after more than 10 years of smoking cessation [43]. Research by Kocer et al. [44] found that retinal superficial and deep capillary densities in the smoking group (*n* = 30) were lower than in the healthy control group (*n* = 31), but no significant difference in RNFL thickness was observed between the two groups. Therefore, further investigation is needed to elucidate the relationship between smoking and RNFL thickness.

This study found that RNFL thickness in various regions of COPD patients was significantly thinner than in healthy controls and correlated with disease severity. Furthermore, it was observed that in patients with mild/moderate COPD, the inferior RNFL was the first to be affected. Research has shown that compared to the superior temporal region, the inferior temporal quadrant exhibits a weaker response to vasodilation and a stronger response to vasoconstriction. This difference leads to varying susceptibilities to vascular dysfunction across different regions. Overall, the findings support our hypothesis that changes in the RNFL of COPD patients may be attributed to reduced retinal vascular density.

This study includes research from multiple countries and regions, resulting in findings that have a certain degree of generalizability. However, most of the included studies are observational, which limits the comparability between groups. Additionally, differences in ethnicity across countries and slight variations in ophthalmologic examination parameters might contribute to the heterogeneity observed. Subgroup analysis by the severity of COPD significantly reduced heterogeneity. Sensitivity analysis identified the study by Wagh et al. [14] as a potential source of heterogeneity in this paper, but no significant clinical heterogeneity was found upon full‐text review. Excluding this study and reconducting the meta‐analysis showed that the results remained stable, indicating that the observed heterogeneity is acceptable. Publication bias tests for outcome indicators with more than 10 studies included did not reveal any bias. Kazantzis et al. [45] found that nasal RNFL thickness decreases in the early stages of COPD but is not related to disease progression. However, this conclusion differs from our study, which may be attributed to our updated search period, more comprehensive and standardized search strategy, and more precise statistical methods. This study also has the following limitations: (1) Despite systematic searches, some gray literature may have been omitted; (2) although COPD patients were strictly diagnosed according to GOLD standards, clinical heterogeneity among patients remains uncontrollable; (3) due to limitations in the included studies, subgroup analysis by smoking status was not possible; and (4) some outcome indicators had too few studies to assess publication bias.

## 5 Conclusion

Existing evidence suggests that COPD patients have significantly lower average RNFL thickness and RNFL thickness in each quadrant compared to healthy individuals, with these changes being associated with disease severity. As COPD develops and progresses, changes in RNFL thickness may initially manifest in the inferior RNFL. Although this mechanism may be related to the dysfunction of retinal microvascular regulation in COPD patients, there is currently limited research in this area, and further prospective, high‐quality studies are needed to verify these findings. This study highlights the potential value of regular RNFL thickness assessments in the comprehensive management of COPD. A reduction in inferior RNFL thickness may provide clues for early detection of COPD, whereas changes in RNFL thickness across different quadrants may help assess disease progression in COPD patients. In recent years, with growing attention to the retinal and optic nerve structures in COPD patients, novel biomarkers such as RNFL are expected to play a significant role in the prevention and long‐term management of COPD complications, offering new insights into the systemic impact of COPD.

## Author Contributions

Yunpeng Xu was responsible for the study design and implementation, data organization, and manuscript writing. Peidong Shi and Xiaoying Liu handled data extraction, statistical analysis, and cross‐checking. Ziyi Jiang conducted the literature review and evaluation. Xunwen Lei and Xue Bai provided guidance on study design and statistical analysis. Jian Liu and Fanqi Wu were in charge of manuscript revision, quality control, and final proofreading.

## Consent

All authors consent to the publication of images, videos, or recordings in this article and agree with its content. Signed consent forms can be provided to the journal's editorial office upon request.

## Conflicts of Interest

The authors declare no conflicts of interest.

## Supporting information


**Table S1** Characterstics of the Included Studies.
**Table S2** Results of Bias Risk Assessment in Observational Cohort Studies and Cross‐Sectional Studies.
**Table S3** Results of Bias Risk Assessment in Included Case–Control Studies.
**Figure S1** Forest plot of the AVERAGE RNFL level between Mild/Moderate COPD Vs HEALTH patients.
**Figure S2** Forest plot of the INFERIOR RNFL level between Mild/Moderate COPD Vs HEALTH patients.
**Figure S3** Forest plot of the NASAL RNFL level between Mild/Moderate COPD Vs HEALTH patients.
**Figure S4** Forest plot of the SUPERIOR RNFL level between Mild/Moderate COPD Vs HEALTH patients.
**Figure S5** Forest plot of the TEMPORAL RNFL level between Mild/Moderate COPD Vs HEALTH patients.
**Figure S6** Forest plot of the AVERAGE RNFL level between Severe COPD Vs HEALTH patients.
**Figure S7** Forest plot of the INFERIOR RNFL level between Severe COPD Vs HEALTH patients.
**Figure S8** Forest plot of the NASAL RNFL level between Severe COPD Vs HEALTH patients.
**Figure S9** Forest plot of the SUPERIOR RNFL level between Severe COPD Vs HEALTH patients.
**Figure S10** Forest plot of the TEMPORAL RNFL level between Severe COPD Vs HEALTH patients.
**Figure S11** Forest plot of the AVERAGE RNFL level between Mild/Moderate COPD Vs Severe COPD patients.
**Figure S12** Forest plot of the INFERIOR RNFL level between Mild/Moderate COPD Vs Severe COPD patients.
**Figure S13** Forest plot of the NASAL RNFL level between Mild/Moderate COPD Vs Severe COPD patients.
**Figure S14** Forest plot of the SUPERIOR RNFL level between Mild/Moderate COPD Vs Severe COPD patients.
**Figure S15** Forest plot of the TEMPORAL RNFL level between Mild/Moderate COPD Vs Severe COPD patients.
**Figure S16** Egger’s Publication Bias Plot for AVERAGE RNFL in COPD Versus Healthy Subjects.
**Figure S17** Egger’s Publication Bias Plot for INFERIOR RNFL in COPD Versus Healthy Subjects.
**Figure S18** Egger’s Publication Bias Plot for NASAL RNFL in COPD Versus Healthy Subjects.
**Figure S19** Egger’s Publication Bias Plot for SUPERIOR RNFL in COPD Versus Healthy Subjects.
**Figure S20** Egger’s Publication Bias Plot for TEMPORAL RNFL in COPD Versus Healthy Subjects.

## Data Availability

The data that support the findings of this study are available in the Supporting Information of this article.
