# Supplementary material for: Changes in Retinal Nerve Fiber Layer Thickness in Patients With Chronic Obstructive Pulmonary Disease: A Systematic Review and Meta‐Analysis
Source: Clin Respir J. 2025 Mar 4;19(3):e70065. doi: 10.1111/crj.70065 (PMC11876992; doi:10.1111/crj.70065)
Supplement: Supplementary file 1 — Table S1 Characterstics of the Included Studies. Table S2 Results of Bias Risk Assessment in Observational Cohort Studies and Cross‐Sectional Studies. Table S3 Results of Bias Risk Assessment in Included Case–Control Studies. Figure S1 Forest plot of the AVERAGE RNFL level between Mild/Moderate COPD Vs HEALTH patients. Figure S2 Forest plot of the INFERIOR RNFL level between Mild/Moderate COPD Vs HEALTH patients. Figure S3 Forest plot of the NASAL RNFL level between Mild/Moderate COPD Vs HEALTH patients. Figure S4 Forest plot of the SUPERIOR RNFL level between Mild/Moderate COPD Vs HEALTH patients. Figure S5 Forest plot of the TEMPORAL RNFL level between Mild/Moderate COPD Vs HEALTH patients. Figure S6 Forest plot of the AVERAGE RNFL level between Severe COPD Vs HEALTH patients. Figure S7 Forest plot of the INFERIOR RNFL level between Severe COPD Vs HEALTH patients. Figure S8 Forest plot of the NASAL RNFL level between Severe COPD Vs HEALTH patients. Figure S9 Forest plot of the SUPERIOR RNFL level between Severe COPD Vs HEALTH patients. Figure S10 Forest plot of the TEMPORAL RNFL level between Severe COPD Vs HEALTH patients. Figure S11 Forest plot of the AVERAGE RNFL level between Mild/Moderate COPD Vs Severe COPD patients. Figure S12 Forest plot of the INFERIOR RNFL level between Mild/Moderate COPD Vs Severe COPD patients. Figure S13 Forest plot of the NASAL RNFL level between Mild/Moderate COPD Vs Severe COPD patients. Figure S14 Forest plot of the SUPERIOR RNFL level between Mild/Moderate COPD Vs Severe COPD patients. Figure S15 Forest plot of the TEMPORAL RNFL level between Mild/Moderate COPD Vs Severe COPD patients. Figure S16 Egger’s Publication Bias Plot for AVERAGE RNFL in COPD Versus Healthy Subjects. Figure S17 Egger’s Publication Bias Plot for INFERIOR RNFL in COPD Versus Healthy Subjects. Figure S18 Egger’s Publication Bias Plot for NASAL RNFL in COPD Versus Healthy Subjects. Figure S19 Egger’s Publication Bias Plot for SUPERIOR RNFL in COPD Versus Heal [file CRJ-19-e70065-s001.docx]

**Supplementary Material**

**Supplementary Search Terms and Strategy**

**English search terms:** “Retinal nerve fibre layer; RNFL” and “Pulmonary Disease, Chronic Obstructive; Chronic Obstructive Lung Disease; Chronic Obstructive Pulmonary Diseases; COAD; COPD; Chronic Obstructive Airway Disease; Chronic Obstructive Pulmonary Disease; Airflow Obstruction, Chronic; Airflow Obstructions, Chronic; Chronic Airflow Obstructions; Chronic Airflow Obstruction; chronic airway obstruction; chronic obstructive bronchopulmonary disease; chronic obstructive lung disorder; chronic obstructive pulmonary disorder; chronic obstructive respiratory disease; chronic pulmonary obstructive disease; chronic pulmonary obstructive disorder; lung chronic obstructive disease; lung disease, chronic obstructive; obstructive chronic lung disease; obstructive chronic pulmonary disease; obstructive lung disease, chronic; pulmonary disorder, chronic obstructive”.

**Chinese** **search terms:** “肺疾病, 慢性阻塞性; 慢性阻塞肺疾病; COAD; 慢性气道阻塞性疾病; 慢性阻塞性肺疾病; 慢性气流阻塞; 慢性气道阻塞; 气道阻塞, 慢性; COPD” and “视网膜神经纤维层; RNFL”。

**Complete search strategy:**

| PubMed | search terms | Numbers |
| --- | --- | --- |
| #1 | "pulmonary disease, chronic obstructive"[MeSH Terms] | 68690 |
| #2 | "Pulmonary Disease, Chronic Obstructive"[Title/Abstract] OR "Chronic Obstructive Lung Disease"[Title/Abstract] OR "Chronic Obstructive Pulmonary Diseases"[Title/Abstract] OR "COAD"[Title/Abstract] OR "COPD"[Title/Abstract] OR "Chronic Obstructive Airway Disease"[Title/Abstract] OR "Chronic Obstructive Pulmonary Disease"[Title/Abstract] OR "Airflow Obstruction, Chronic"[Title/Abstract] OR "Airflow Obstructions, Chronic"[Title/Abstract] OR "Chronic Airflow Obstructions"[Title/Abstract] OR "Chronic Airflow Obstruction"[Title/Abstract] OR "chronic airway obstruction"[Title/Abstract] OR "chronic obstructive bronchopulmonary disease"[Title/Abstract] OR "chronic obstructive lung disorder"[Title/Abstract] OR "chronic obstructive pulmonary disorder"[Title/Abstract] OR "chronic obstructive respiratory disease"[Title/Abstract] OR "chronic pulmonary obstructive disease"[Title/Abstract] OR "chronic pulmonary obstructive disorder"[Title/Abstract] OR "lung chronic obstructive disease"[Title/Abstract] OR "lung disease, chronic obstructive"[Title/Abstract] OR "obstructive chronic lung disease"[Title/Abstract] OR "obstructive chronic pulmonary disease"[Title/Abstract] OR "obstructive lung disease, chronic"[Title/Abstract] OR "pulmonary disorder, chronic obstructive"[Title/Abstract] | 89896 |
| #3 | "retinal nerve fibre layer"[Title/Abstract] OR "RNFL"[Title/Abstract] | 5895 |
| #4 | #1 OR #2 | 111122 |
| #5 | #3 AND #4 | 12 |
|  |  |  |
| Embase | search terms | Numbers |
| #1 | 'chronic obstructive lung disease'/exp | 183679 |
| #2 | 'pulmonary disease, chronic obstructive':ab,ti OR 'chronic obstructive lung disease':ab,ti OR 'chronic obstructive pulmonary diseases':ab,ti OR 'coad':ab,ti OR 'copd':ab,ti OR 'chronic obstructive airway disease':ab,ti OR 'chronic obstructive pulmonary disease':ab,ti OR 'airflow obstruction, chronic':ab,ti OR 'airflow obstructions, chronic':ab,ti OR 'chronic airflow obstructions':ab,ti OR 'chronic airflow obstruction':ab,ti OR 'chronic airway obstruction':ab,ti OR 'chronic obstructive bronchopulmonary disease':ab,ti OR 'chronic obstructive lung disorder':ab,ti OR 'chronic obstructive pulmonary disorder':ab,ti OR 'chronic obstructive respiratory disease':ab,ti OR 'chronic pulmonary obstructive disease':ab,ti OR 'chronic pulmonary obstructive disorder':ab,ti OR 'lung chronic obstructive disease':ab,ti OR 'lung disease, chronic obstructive':ab,ti OR 'obstructive chronic lung disease':ab,ti OR 'obstructive chronic pulmonary disease':ab,ti OR 'obstructive lung disease, chronic':ab,ti OR 'pulmonary disorder, chronic obstructive':ab,ti | 149728 |
| #3 | 'retinal nerve fibre layer':ab,ti OR 'rnfl':ab,ti | 9044 |
| #4 | #1 OR #2 | 212142 |
| #5 | #3 AND #4 | 12 |
|  |  |  |
| Cochrane | search terms | Numbers |
| #1 | MeSH descriptor: [Pulmonary Disease, Chronic Obstructive] explode all trees | 8273 |
| #2 | (Pulmonary Disease, Chronic Obstructive):ti,ab,kw OR (Chronic Obstructive Lung Disease):ti,ab,kw OR (Chronic Obstructive Pulmonary Diseases):ti,ab,kw OR (COAD):ti,ab,kw OR (COPD):ti,ab,kw OR (Chronic Obstructive Airway Disease):ti,ab,kw OR (Chronic Obstructive Pulmonary Disease):ti,ab,kw OR (Airflow Obstruction, Chronic):ti,ab,kw OR (Airflow Obstructions, Chronic):ti,ab,kw OR (Chronic Airflow Obstructions):ti,ab,kw OR (Chronic Airflow Obstruction):ti,ab,kw OR (chronic airway obstruction):ti,ab,kw OR (chronic obstructive bronchopulmonary disease):ti,ab,kw OR (chronic obstructive lung disorder):ti,ab,kw OR (chronic obstructive pulmonary disorder):ti,ab,kw OR (chronic obstructive respiratory disease):ti,ab,kw OR (chronic pulmonary obstructive disease):ti,ab,kw OR (chronic pulmonary obstructive disorder):ti,ab,kw OR (lung chronic obstructive disease):ti,ab,kw OR (lung disease, chronic obstructive):ti,ab,kw OR (obstructive chronic lung disease):ti,ab,kw OR (obstructive chronic pulmonary disease):ti,ab,kw OR (obstructive lung disease, chronic):ti,ab,kw OR (pulmonary disorder, chronic obstructive):ti,ab,kw | 25392 |
| #3 | (retinal nerve fibre layer):ti,ab,kw OR (RNFL):ti,ab,kw | 722 |
| #4 | #1 OR #2 | 25738 |
| #5 | #4 AND #3 | 1 |
|  |  |  |
| Web of science | search terms | Numbers |
| #1 | TS=(Pulmonary Disease, Chronic Obstructive) OR TS=(Chronic Obstructive Lung Disease) OR TS=(Chronic Obstructive Pulmonary Diseases) OR TS=(COAD) OR TS=(COPD) OR TS=(Chronic Obstructive Airway Disease) OR TS=(Chronic Obstructive Pulmonary Disease) OR TS=(Airflow Obstruction, Chronic) OR TS=(Airflow Obstructions, Chronic) OR TS=(Chronic Airflow Obstructions) OR TS=(Chronic Airflow Obstruction) OR TS=(chronic airway obstruction) OR TS=(chronic obstructive bronchopulmonary disease) OR TS=(chronic obstructive lung disorder) OR TS=(chronic obstructive pulmonary disorder) OR TS=(chronic obstructive respiratory disease) OR TS=(chronic pulmonary obstructive disease) OR TS=(chronic pulmonary obstructive disorder) OR TS=(lung chronic obstructive disease) OR TS=(lung disease, chronic obstructive) OR TS=(obstructive chronic lung disease) OR TS=(obstructive chronic pulmonary disease) OR TS=(obstructive lung disease, chronic) OR TS=(pulmonary disorder, chronic obstructive) | 176849 |
| #2 | TS=(retinal nerve fibre layer) OR TS=(RNFL) | 18281 |
| #3 | #2 AND #1 | 20 |
|  |  |  |
| CNKI(China National Knowledge Infrastructure) | search terms | Numbers |
| #1 | TI='肺疾病, 慢性阻塞性' OR TI='慢性阻塞肺疾病' OR TI='COAD; 慢性气道阻塞性疾病' OR TI='慢性阻塞性肺疾病' OR TI='慢性气流阻塞' OR TI='慢性气道阻塞' OR TI='气道阻塞, 慢性' OR TI='COPD' | 61200 |
| #2 | TI='视网膜神经纤维层' OR TI='RNFL' | 530 |
| #3 | #1 AND #2 | 2 |
|  |  |  |
| Wanfang Data | search terms | Numbers |
| #1 | 主题:(肺疾病, 慢性阻塞性) or 主题:(慢性阻塞肺疾病) or 主题:(COAD) or 主题:(慢性气道阻塞性疾病) or 主题:(慢性阻塞性肺疾病) or 主题:(慢性气流阻塞) or 主题:(慢性气道阻塞) or 主题:(气道阻塞, 慢性) or 主题:(COPD) | 141087 |
| #2 | 主题:(视网膜神经纤维层) or 主题:(RNFL) | 6504 |
| #3 | #1 AND #2 | 9 |
|  |  |  |
| VIP (Very Important Paper) | search terms | Numbers |
| #1 | M=(肺疾病, 慢性阻塞性 OR 慢性阻塞肺疾病 OR COAD OR 慢性气道阻塞性疾病 OR 慢性阻塞性肺疾病 OR 慢性气流阻塞 OR 慢性气道阻塞 OR 气道阻塞, 慢性 OR COPD) | 74868 |
| #2 | M=(视网膜神经纤维层 OR RNFL) | 819 |
| #3 | #1 AND #2 | 1 |
|  |  |  |
| CBM (China Biology Medicine disc) | search terms | Numbers |
| #1 | ("肺疾病, 慢性阻塞性"[常用字段:智能] OR "慢性阻塞肺疾病"[常用字段:智能] OR "COAD"[常用字段:智能] OR "慢性气道阻塞性疾病"[常用字段:智能] OR "慢性阻塞性肺疾病"[常用字段:智能] OR "慢性气流阻塞"[常用字段:智能] OR "慢性气道阻塞"[常用字段:智能] OR "气道阻塞, 慢性"[常用字段:智能] OR "COPD"[常用字段:智能]) | 88601 |
| #2 | ("视网膜神经纤维层"[常用字段:智能] OR "RNFL"[常用字段:智能]) | 1657 |
| #3 | #1 AND #2 | 1 |

A supplementary search on Google Scholar yielded 9 documents.

**Supplementary Table 1** **Characterstics of the Included Studies**

| First author | Year | Country | Age, years (Mean±SD) | Gender, n (male/female) | Sample size,n | Body mass index（BMI, kg/m2） | Intraocular pressure(IOP, mmHg） |
| --- | --- | --- | --- | --- | --- | --- | --- |
| Ahmed NO^19^ | 2021 | Egypt | COPD group: 49.66 ± 4.77 HEALTH group: 58.68 ± 1.58 | COPD group: 42/8 HEALTH group: 24/26 | COPD group: 50 HEALTH group: 50 | — | — |
| Alim S^26^ | 2019 | Turkey | COPD group: 61.69 ± 7.20 HEALTH group: 58.19 ± 8.94 | COPD group: 26/0 HEALTH group: 26/0 | COPD group: 26 HEALTH group: 26 | — | — |
| Alkan AA^23^ | 2021 | Turkey | COPD group: 60.50 ± 8.20 HEALTH group: 60.90 ± 7.70 | COPD group: 25/10 HEALTH group: 25/10 | COPD group: 50 HEALTH group: 50 | — | — |
| Ghee YT^18^ | 2017 | Malaysia | COPD group: 68.27 ± 6.62 HEALTH group: 69.73 ± 5.14 | COPD group: 55/16 HEALTH group: 53/18 | COPD group: 71 HEALTH group: 71 | — | — |
| Gok M^27^ | 2018 | Turkey | COPD group: 63.34 ± 9.37 HEALTH group: 62.03 ± 11.26 | COPD group: 59/20 HEALTH group: 50/21 | COPD group: 79 HEALTH group: 71 | COPD group: 23.05 ± 1.60 HEALTH group: 22.54 ± 1.52 | — |
| Kocamış Ö^15^ | 2018 | Turkey | Mild/Moderate COPD group: 64.40 ± 8.60 Severe COPD group: 64.10 ± 7.30 HEALTH group：65.80 ± 7.10 | — | Mild/Moderate COPD group: 30 Severe COPD group: 30 HEALTH group: 23 | — | — |
| Kurtul BE^22^ | 2022 | Turkey | Mild/Moderate COPD group: 58.70 ± 11.10 Severe COPD group: 59.60 ± 13.80 HEALTH group: 55.10 ± 8.00 | Mild/Moderate COPD group: 21/1 Severe COPD group: 18/0 HEALTH group: 29/1 | Mild/Moderate COPD group: 22 Severe COPD group: 18 HEALTH group: 30 | Mild/Moderate COPD group: 27.80 ± 4.80 Severe COPD group: 27.80 ± 4.80 HEALTH group: 27.00 ± 2.90 | Mild/Moderate COPD group: 16.40 ± 4.10 Severe COPD group: 17.00 ± 4.60 HEALTH group: 16.30 ± 1.50 |
| Kurumoğlu İncekalan T^21^ | 2023 | India | Mild COPD group: 59.72 ± 8.67 Moderate COPD group: 57.90 ± 8.80 Severe COPD group: 53.53 ± 8.21 HEALTH group: 58.48 ± 8.54 | Mild COPD group: 17/5 Moderate COPD group: 15/8 Severe COPD group: 16/5 HEALTH group: 40/14 | Mild COPD group: 22 Moderate COPD group: 23 Severe COPD group: 21 HEALTH group: 54 | Mild COPD group: 25.33 ± 2.81 Moderate COPD group: 27.5 ± 3.52 Severe COPD group: 27.08 ± 5.09 HEALTH group: 25.99 ± 7.03 | — |
| Lee JS^20^ | 2023 | Canada | COPD group: 75.90 ± 3.70 HEALTH group: 72.00 ± 4.70 | COPD group: 21/9 HEALTH group: 7/33 | COPD group: 30 HEALTH group: 40 | COPD group: 25.00 ± 2.70 HEALTH group: 24.10 ± 3.20 | COPD group: 12.80 ± 2.10 HEALTH group: 13.40 ± 2.50 |
| Li Lv^28^ | 2018 | Korea | COPD group: 71.33 ± 8.89 HEALTH group: 68.10 ± 9.91 | COPD group: 19/11 HEALTH group: 17/13 | COPD group: 30 HEALTH group: 30 | COPD group: 23.17 ± 1.53 HEALTH group: 22.26 ± 1.98 | — |
| Ogan N^25^ | 2020 | Turkey | Mild/Moderate COPD group: 62.10 ± 12.50 Severe COPD group: 69.50 ± 11.20 HEALTH group: 67.00 ± 6.70 | Mild/Moderate COPD group: 10/4 Severe COPD group: 28/6 HEALTH group: 17/23 | Mild/Moderate COPD group: 14 Severe COPD group: 34 HEALTH group: 40 | Mild/Moderate COPD group: 27.00 ± 5.80  Severe COPD group: 26.10 ± 6.00 | — |
| Ozcimen M^29^ | 2016 | Turkey | COPD group: 65.60 ± 8.08  HEALTH group: 63.02 ± 5.62 | COPD group: 45/28 HEALTH group: 29/21 | COPD group: 73 HEALTH group: 50 | COPD group: 22.90 ± 1.75  HEALTH group: 22.30 ± 1.81 | COPD group: 15.90 ± 3.43  HEALTH group: 14.72 ± 3.37 |
| Ozer PA^10^ | 2020 | Turkey | COPD group: 67.49 ± 11.35 HEALTH group: 66.69 ± 6.62 | COPD group: 39/16 HEALTH group: 20/28 | COPD group: 55 HEALTH group: 48 | — | — |
| Ugurlu E^8^ | 2018 | Turkey | COPD group: 63.80 ± 9.50 HEALTH group: 61.60 ± 8.50 | — | — | — | COPD group: 13.60 ± 2.80  HEALTH group: 14.10 ± 1.80 |
| Wagh V^14^ | 2022 | India | Mild/Moderate COPD group: 56.20 ± 4.22  Severe COPD group: 57.88 ± 4.83  HEALTH group: 57.28 ± 4.46 | Mild/Moderate COPD group: 28/12 Severe COPD group: 25/15 HEALTH group: 31/9 | Mild/Moderate COPD group: 40  Severe COPD group: 40 HEALTH group: 40 | — | Mild/Moderate COPD group: 13.20 ± 2.51  Severe COPD group: 14.33 ± 4.06 HEALTH group: 12.15 ± 2.26 |

**Supplementary Table 1 (Continued): Characteristics of the Included Studies**

| First author | Year | Axial length (mm) | FEV1(%) | FEV1/FVC(%) | Arterial oxygen saturation(SaO2, %) | Disease duration (years) | Study types |
| --- | --- | --- | --- | --- | --- | --- | --- |
| Ahmed NO^19^ | 2021 | — | COPD group: 54.29 ± 20.68 | — | COPD group: 89.76 ± 10.44 | — | Case-control study |
| Alim S^26^ | 2019 | COPD group: 22.00 ± 2.23 HEALTH group: 23.00 ± 2.25 | — | COPD group: 53.18 ± 10.55 HEALTH group: 78.90 ± 4.11 | — | — | Case-control study |
| Alkan AA^23^ | 2021 | COPD group: 22.60 ± 0.90 HEALTH group: 22.40 ± 0.90 | COPD group: 44.30 ± 13.10 HEALTH group: — | COPD group: 62.30 ± 7.10 | COPD group: 89.10 ± 3.40 HEALTH group: 97.90 ± 0.80 | COPD group: 10.30 ± 4.60 | Cross‑sectional study |
| Ghee YT^18^ | 2017 | — | — | — | — | COPD group: 8.01 ± 4.46 | Case-control study |
| Gok M^27^ | 2018 | — | — | — | — | COPD group: 10.42 ± 4.11 | Cross‑sectional study |
| Kocamış Ö^15^ | 2018 | — | — | Mild/Moderate COPD group: 71.70 ± 13.10 Severe COPD group: 63.90 ± 14.80 HEALTH group: 90.20 ± 4.20 | Mild/Moderate COPD group: 94.20 ± 4.40 Severe COPD group: 89.30 ± 8.50 HEALTH group: 98.00 ± 0.90 | — | Cross‑sectional study |
| Kurtul BE^22^ | 2022 | — | Mild/Moderate COPD group: 64.40 ± 8.60 Severe COPD group: 64.10 ± 7.30 | Mild/Moderate COPD group: 67.80 ± 9.90 Severe COPD group: 59.50 ± 10.70 | Mild/Moderate COPD group: 96.00 ± 1.10 Severe COPD group: 93.20 ± 4.10 | Mild/Moderate COPD group: 3.00 ± 2.97 Severe COPD group: 4.00 ± 6.67 | Cross‑sectional study |
| Kurumoğlu İncekalan T^21^ | 2023 | Mild COPD group: 22.50 ± 0.50 Moderate COPD group: 96.00 ± 1.10 Severe COPD group: 23.10 ± 0.90 HEALTH group: 22.80 ± 0.90 | — | Mild COPD group: 52.33 ± 11.74 Moderate COPD group: 62.50 ± 4.21 Severe COPD group: 68.53 ± 8.38 HEALTH group: 80.40 ± 5.69 | Mild COPD group: 89.61 ± 2.70 Moderate COPD group: 94.55 ± 1.43 Severe COPD group: 95.79 ± 2.59 HEALTH group: 98.63 ± 1.29 | Mild COPD group: 17.06 ± 6.59 Moderate COPD group: 12.95 ± 6.37 Severe COPD group: 8.21 ± 4.66 | Cross‑sectional study |
| Lee JS^20^ | 2023 | — | COPD group: 75.30 ± 17.70 HEALTH group: 98.70 ± 10.30 | COPD group: 62.30 ± 7.90 HEALTH group: 76.60 ± 4.00 | — | — | Cohort study |
| Li Lv^28^ | 2018 | — | COPD group: 66.10 ± 2.27 HEALTH group: 103.68 ± 1.98 | — | — | — | Cross‑sectional study |
| Ogan N^25^ | 2020 | — | Mild/Moderate COPD group: 55.50 ± 20.00  Severe COPD group: 45.40 ± 18.80 | Mild/Moderate COPD group: 62.60 ± 13.00  Severe COPD group: 56.40 ± 16.90 | Mild/Moderate COPD group: 95.5 (94.7–98)* Severe COPD group: 88.8 (82.6–98)* | Mild/Moderate COPD group: 5 (1–15)* Severe COPD group: 6 (2–20)* | Case-control study |
| Ozcimen M^29^ | 2016 | COPD group: 22.40 ± 1.44  HEALTH group: 22.89 ± 1.28 | — | — | — | COPD group: 11.98 ± 6.19 | Cross‑sectional study |
| Ozer PA^10^ | 2020 | — | — | — | — | COPD group ≥ 5 years | Case-control study |
| Ugurlu E^8^ | 2018 | — | COPD group: 45.40 ± 16.20 | — | — | — | Cross‑sectional study |
| Wagh V^14^ | 2022 | — | Mild/Moderate COPD group: 69.95 ± 10.88  Severe COPD group: 34.63 ± 11.27 | Mild/Moderate COPD group: 0.62 ± 0.06  Severe COPD group: 0.55 ± 0.11 | — | — | Case-control study |

**Supplementary Table 2 Results of Bias Risk Assessment in Observational Cohort Studies and Cross-Sectional Studies**

| First author | Year | ① | ② | ③ | ④ | ⑤ | ⑥ | ⑦ | ⑧ | ⑨ | ⑩ | ⑪ | ⑫ | ⑬ | ⑭ | quality grade |
| --- | --- | --- | --- | --- | --- | --- | --- | --- | --- | --- | --- | --- | --- | --- | --- | --- |
| Alkan AA^23^ | 2021 | Yes | Yes | Yes | Yes | Yes | Yes | Yes | No | Yes | No | Yes | Yes | No | Yes | Good |
| Gok M^27^ | 2018 | Yes | Yes | Yes | Yes | Yes | Yes | Unclear | Yes | Yes | No | Yes | Unclear | No | No | Good |
| Kocamış Ö^15^ | 2018 | Yes | Yes | Yes | Yes | Yes | Yes | Unclear | Yes | Yes | No | Yes | Unclear | No | No | Good |
| Kurtul BE^22^ | 2022 | Yes | Yes | Yes | Yes | Yes | Yes | Unclear | Yes | Yes | No | Yes | Unclear | No | No | Good |
| Kurumoğlu İncekalan T^21^ | 2023 | Yes | Yes | Yes | Yes | Yes | Yes | No | Yes | Yes | No | Yes | Unclear | No | No | Good |
| Lee JS^20^ | 2023 | Yes | Yes | Yes | Yes | Yes | Yes | No | No | Yes | No | Yes | Unclear | No | No | Fair |
| Li Lv^28^ | 2018 | Yes | Yes | Yes | Yes | Yes | Yes | Yes | No | Yes | No | Yes | Unclear | No | No | Good |
| Ozcimen M^29^ | 2016 | Yes | Yes | Yes | Yes | Yes | Yes | Unclear | No | Yes | No | Yes | Unclear | No | No | Good |
| Ugurlu E^8^ | 2018 | Yes | Yes | Yes | Yes | Yes | Yes | Unclear | No | Yes | No | Yes | Unclear | No | No | Good |
| Note: ①Was the research question or objective in this paper clearly stated?; ②Was the study population clearly specified and defined?; ③Was the study population clearly specified and defined?; ④Were all the subjects selected or recruited from the same or similar populations (including the same time period)? Were inclusion and exclusion criteria for being in the study prespecified and applied uniformly to all participants?; ⑤Was a sample size justification, power description, or variance and effect estimates provided?; ⑥For the analyses in this paper, were the exposure(s) of interest measured prior to the outcome(s) being measured?; ⑦Was the timeframe sufficient so that one could reasonably expect to see an association between exposure and outcome if it existed?; ⑧For exposures that can vary in amount or level, did the study examine different levels of the exposure as related to the outcome (e.g., categories of exposure, or exposure measured as continuous variable)?; ⑨Were the exposure measures (independent variables) clearly defined, valid, reliable, and implemented consistently across all study participants?;⑩Was the exposure(s) assessed more than once over time?; ⑪Were the outcome measures (dependent variables) clearly defined, valid, reliable, and implemented consistently across all study participants?; ⑫Were the outcome assessors blinded to the exposure status of participants?; ⑬Was loss to follow-up after baseline 20% or less?; ⑭Were key potential confounding variables measured and adjusted statistically for their impact on the relationship between exposure(s) and outcome(s)? | | | | | | | | | | | | | | | | |

**Supplementary Table 3 Results of Bias Risk Assessment in Included Case-Control Studies**

| First author | Year | ① | ② | ③ | ④ | ⑤ | ⑥ | ⑦ | ⑧ | ⑨ | ⑩ | ⑪ | ⑫ | quality grade |
| --- | --- | --- | --- | --- | --- | --- | --- | --- | --- | --- | --- | --- | --- | --- |
| Ahmed NO^19^ | 2021 | Yes | Yes | Yes | Yes | Yes | Yes | Unclear | Yes | Yes | Yes | Unclear | No | Good |
| Alim S^26^ | 2019 | Yes | Yes | Yes | Yes | Yes | Yes | Unclear | Unclear | Yes | Yes | Unclear | No | Good |
| Ghee YT^18^ | 2017 | Yes | Yes | Yes | Yes | Yes | Yes | Unclear | Yes | Yes | Yes | Unclear | No | Good |
| Ogan N^25^ | 2020 | Yes | Yes | Yes | Yes | Yes | Yes | Unclear | Yes | Yes | Yes | Unclear | No | Good |
| Ozer PA^10^ | 2020 | Yes | Yes | Yes | Yes | Yes | Yes | Unclear | Yes | Yes | Yes | Unclear | No | Good |
| Wagh V^14^ | 2022 | Yes | Yes | Yes | Yes | Yes | Yes | Unclear | Yes | Yes | Yes | Unclear | No | Good |
| Note: ①Was the research question or objective in this paper clearly stated and appropriate?; ②Was the study population clearly specified and defined?; ③Did the authors include a sample size justification?; ④Were controls selected or recruited from the same or similar population that gave rise to the cases (including the same timeframe)?; ⑤Were the definitions, inclusion and exclusion criteria, algorithms or processes used to identify or select cases and controls valid, reliable, and implemented consistently across all study participants?; ⑥Were the cases clearly defined and differentiated from controls?; ⑦If less than 100 percent of eligible cases and/or controls were selected for the study, were the cases and/or controls randomly selected from those eligible?; ⑧Was there use of concurrent controls?; ⑨Were the investigators able to confirm that the exposure/risk occurred prior to the development of the condition or event that defined a participant as a case?; ⑩Were the measures of exposure/risk clearly defined, valid, reliable, and implemented consistently (including the same time period) across all study participants?; ⑪Were the assessors of exposure/risk blinded to the case or control status of participants?; ⑫Were key potential confounding variables measured and adjusted statistically in the analyses? If matching was used, did the investigators account for matching during study analysis? | | | | | | | | | | | | | | |


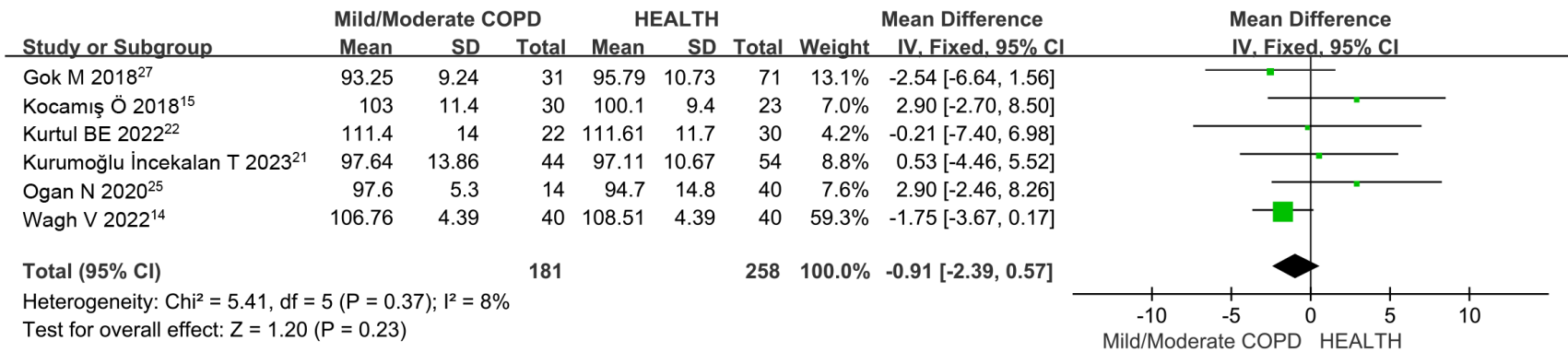


**Supplementary Figure 1** Forest plot of the AVERAGE RNFL level between Mild/Moderate COPD Vs HEALTH patients


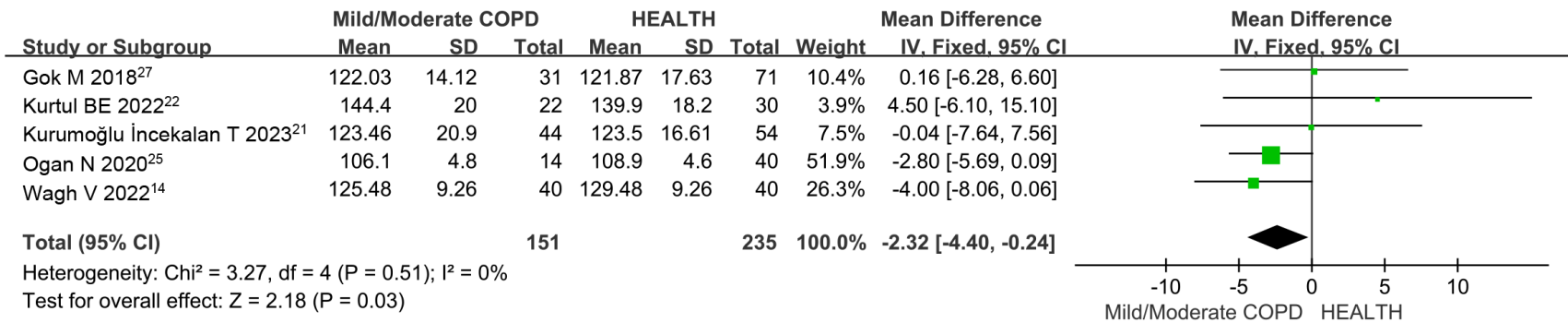


**Supplementary Figure 2** Forest plot of the INFERIOR RNFL level between Mild/Moderate COPD Vs HEALTH patients


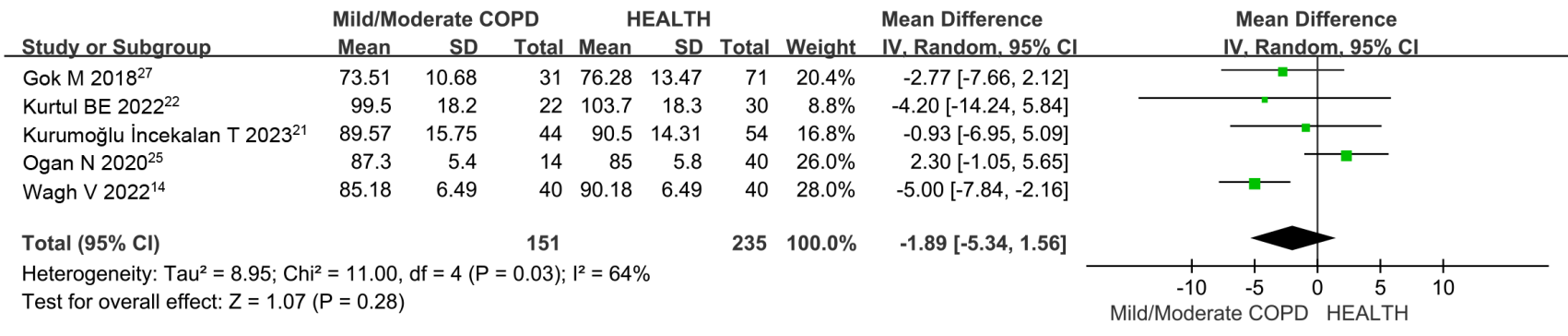


**Supplementary Figure 3** Forest plot of the NASAL RNFL level between Mild/Moderate COPD Vs HEALTH patients


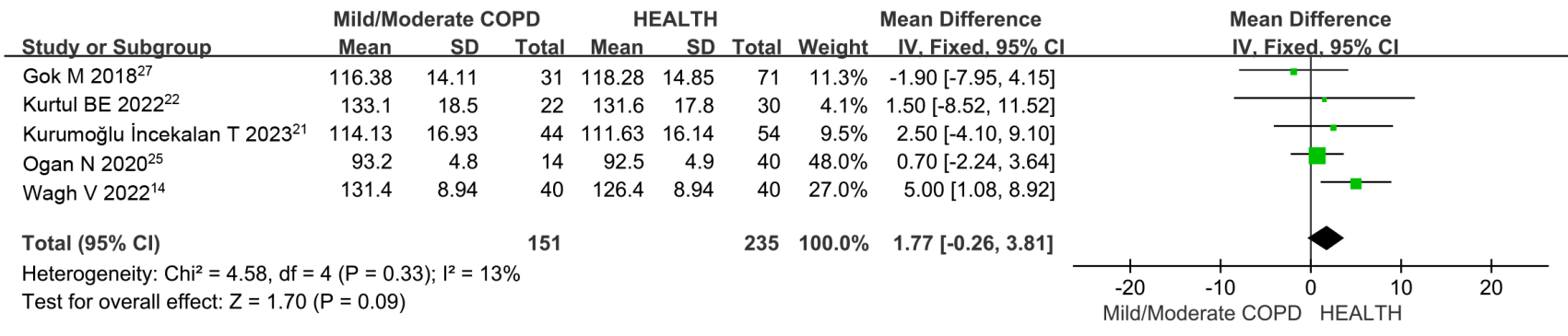


**Supplementary Figure 4** Forest plot of the SUPERIOR RNFL level between Mild/Moderate COPD Vs HEALTH patients


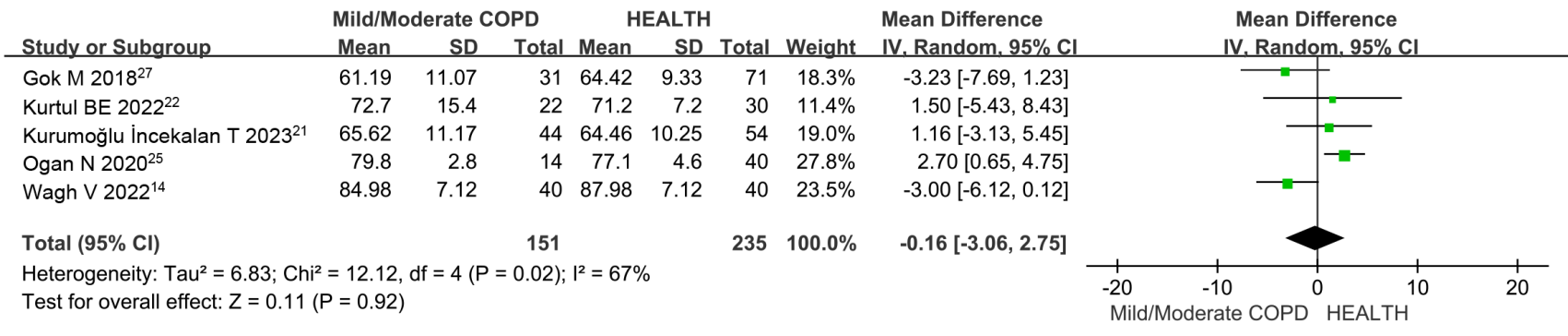


**Supplementary Figure 5** Forest plot of the TEMPORAL RNFL level between Mild/Moderate COPD Vs HEALTH patients


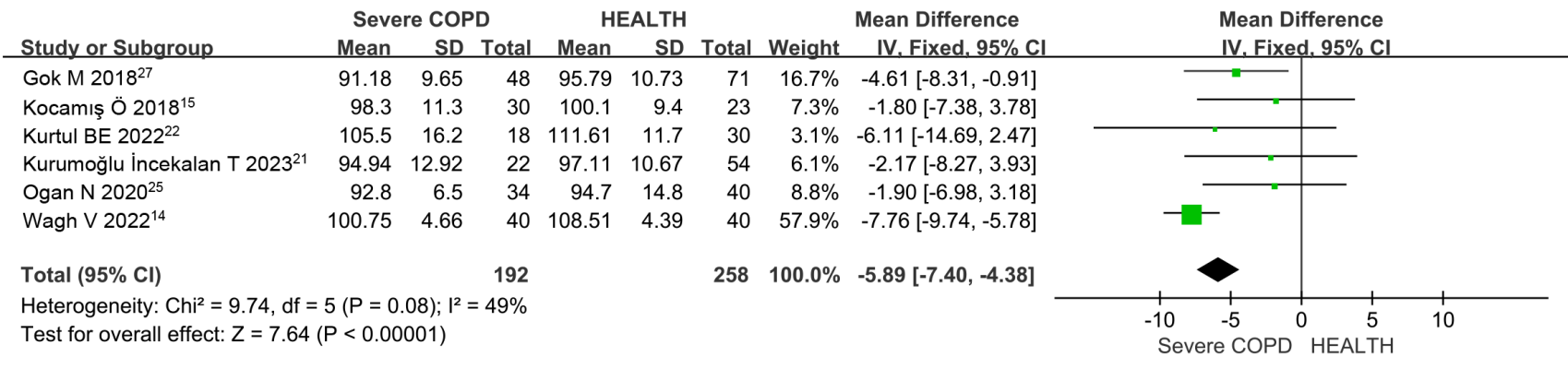


**Supplementary Figure 6** Forest plot of the AVERAGE RNFL level between Severe COPD Vs HEALTH patients


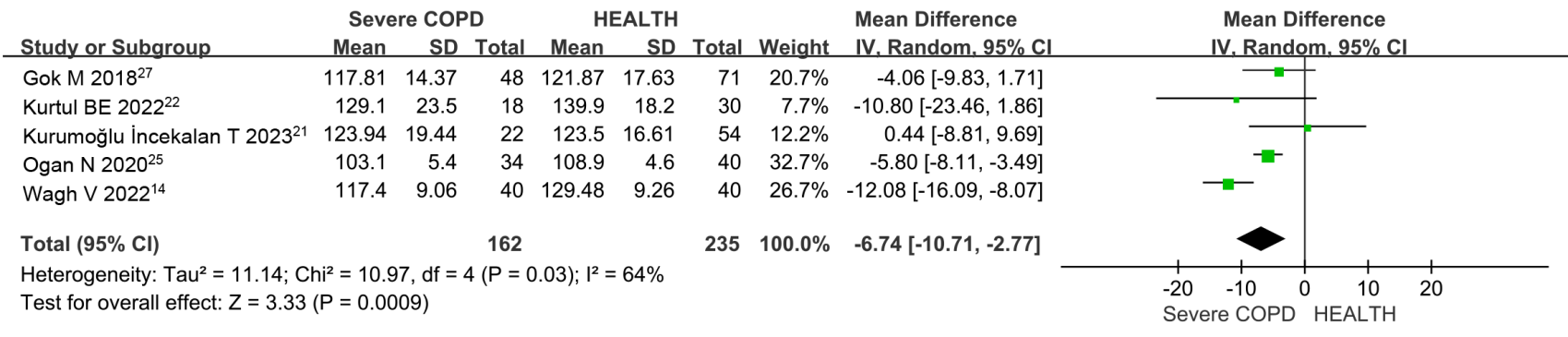


**Supplementary Figure 7** Forest plot of the INFERIOR RNFL level between Severe COPD Vs HEALTH patients


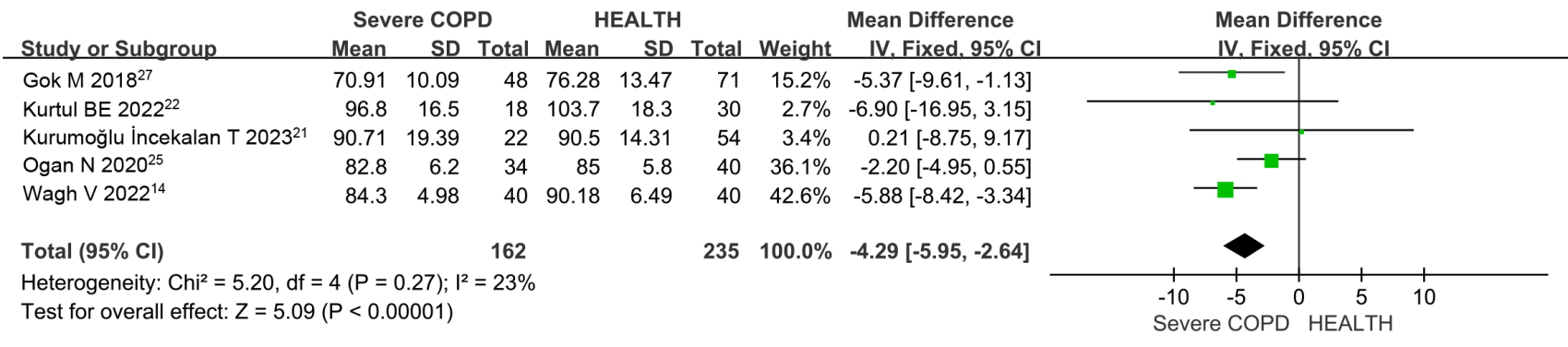


**Supplementary Figure 8** Forest plot of the NASAL RNFL level between Severe COPD Vs HEALTH patients


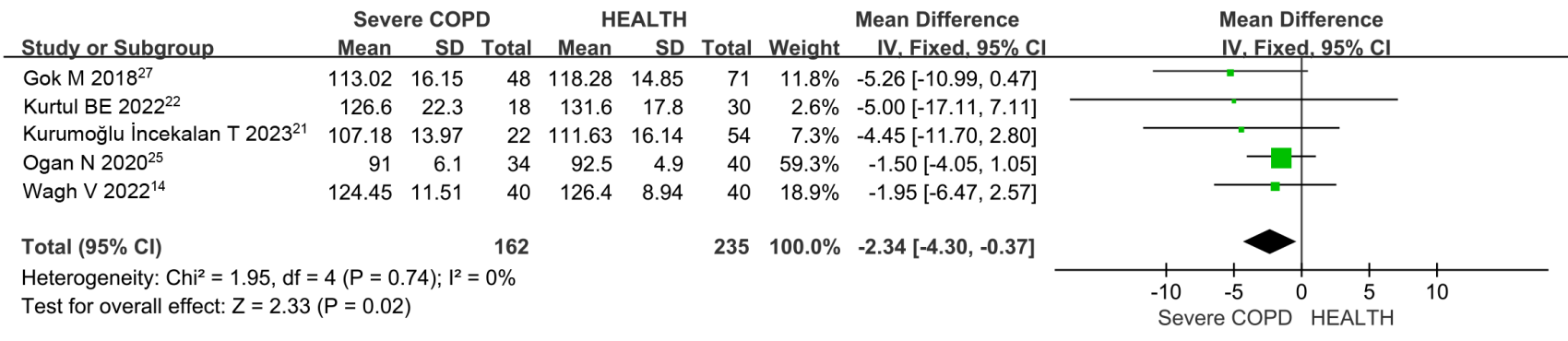


**Supplementary Figure 9** Forest plot of the SUPERIOR RNFL level between Severe COPD Vs HEALTH patients


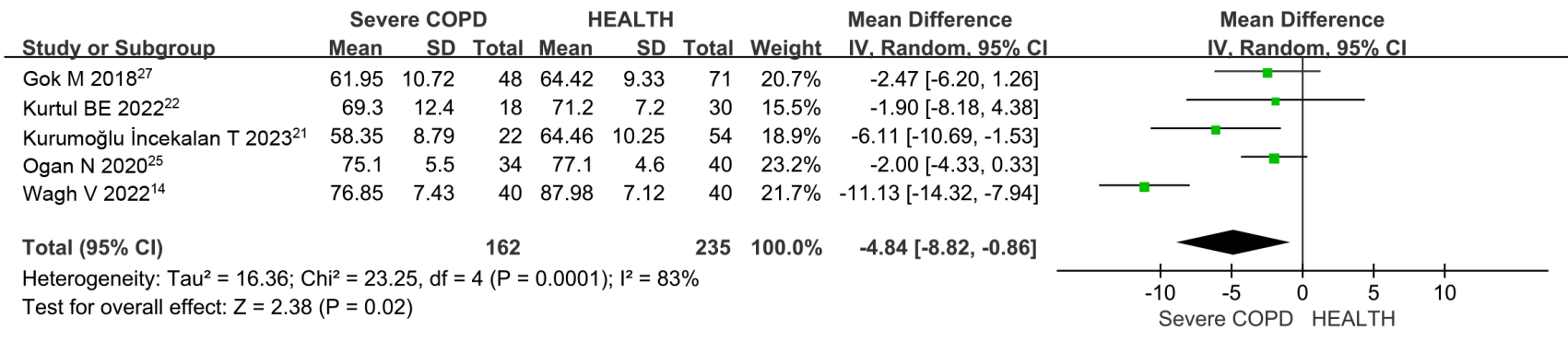


**Supplementary Figure 10** Forest plot of the TEMPORAL RNFL level between Severe COPD Vs HEALTH patients


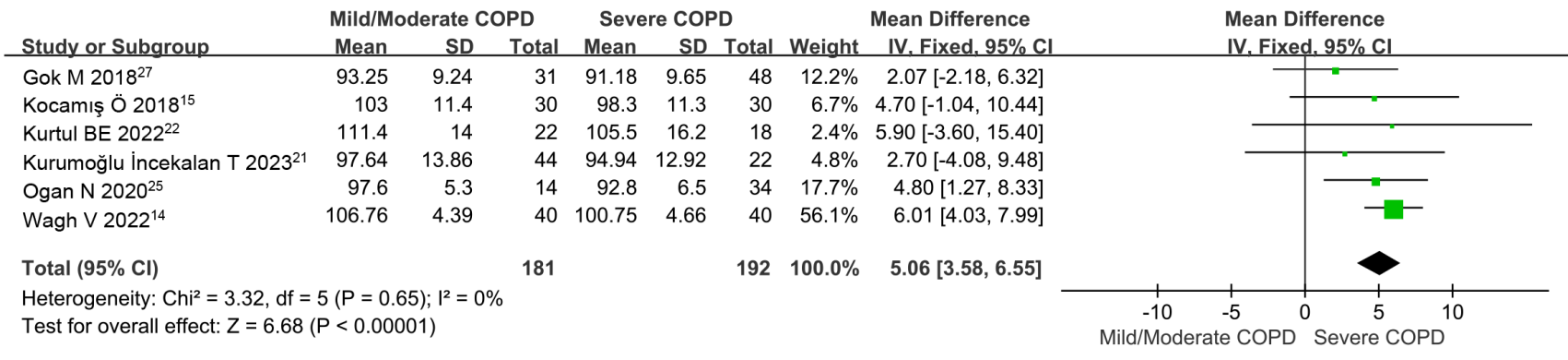


**Supplementary Figure 11** Forest plot of the AVERAGE RNFL level between Mild/Moderate COPD Vs Severe COPD patients


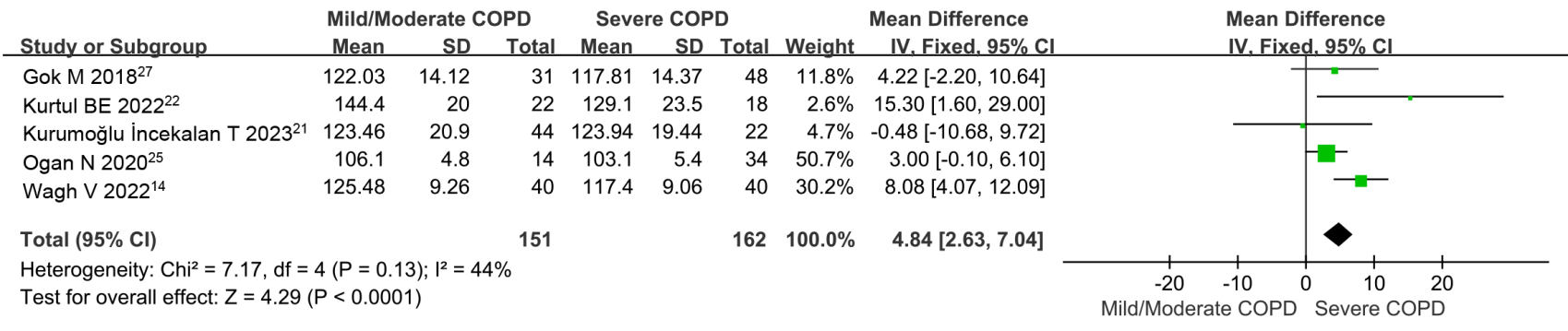


**Supplementary Figure 12** Forest plot of the INFERIOR RNFL level between Mild/Moderate COPD Vs Severe COPD patients


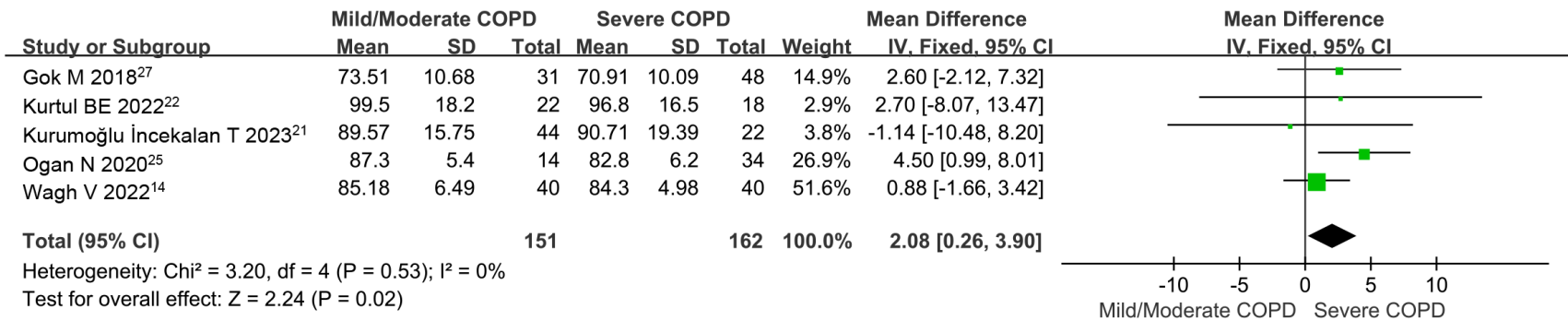


**Supplementary Figure 13** Forest plot of the NASAL RNFL level between Mild/Moderate COPD Vs Severe COPD patients


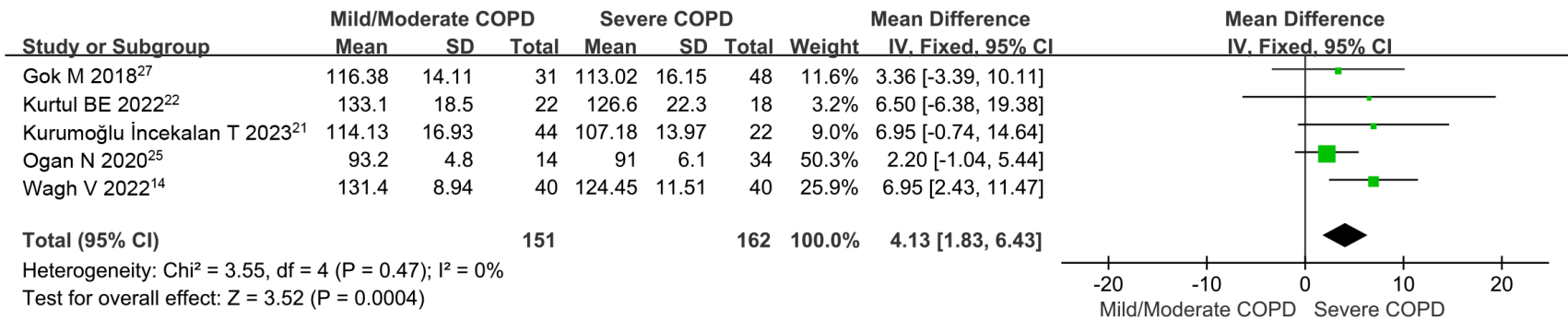


**Supplementary Figure 14** Forest plot of the SUPERIOR RNFL level between Mild/Moderate COPD Vs Severe COPD patients


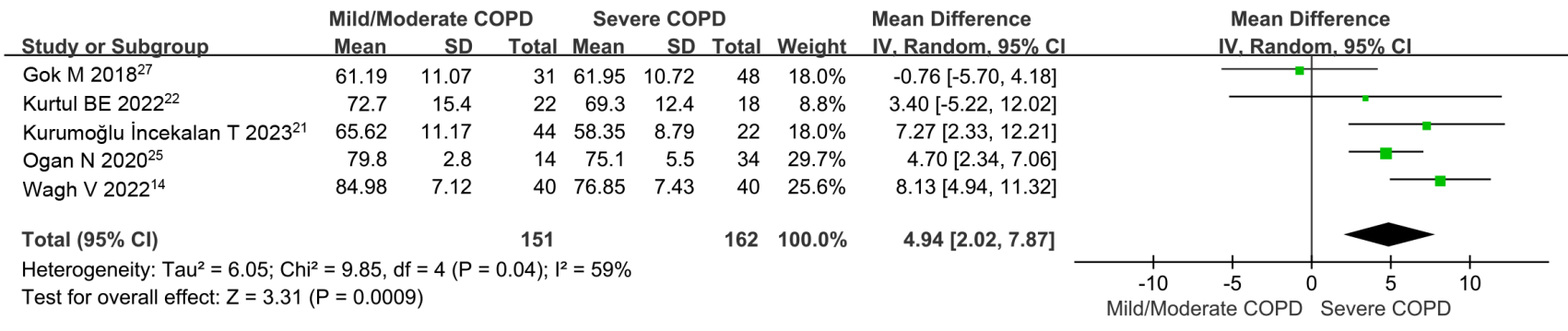


**Supplementary Figure 15** Forest plot of the TEMPORAL RNFL level between Mild/Moderate COPD Vs Severe COPD patients


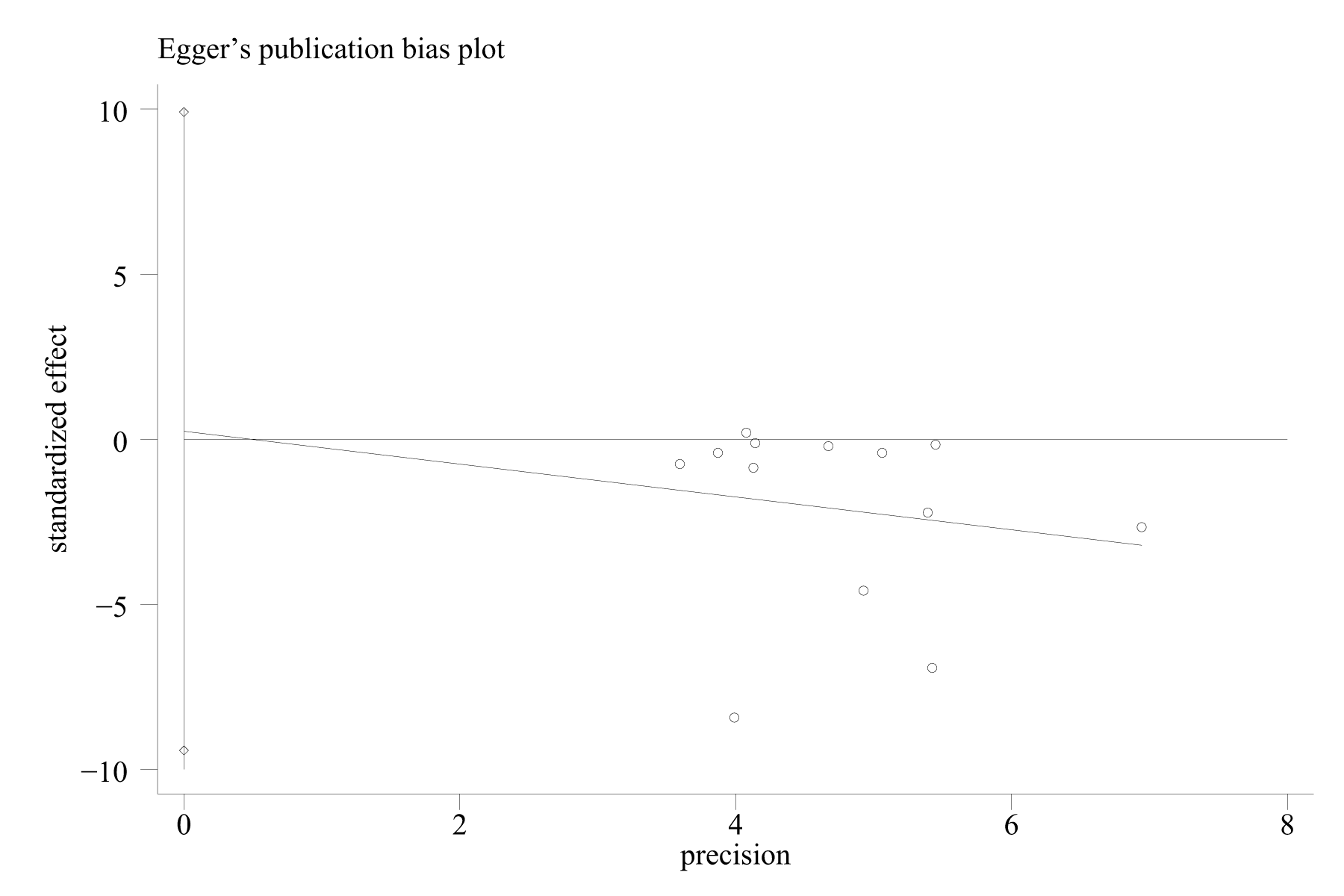


**Supplementary Figure 16** Egger's Publication Bias Plot for AVERAGE RNFL in COPD Versus Healthy Subjects


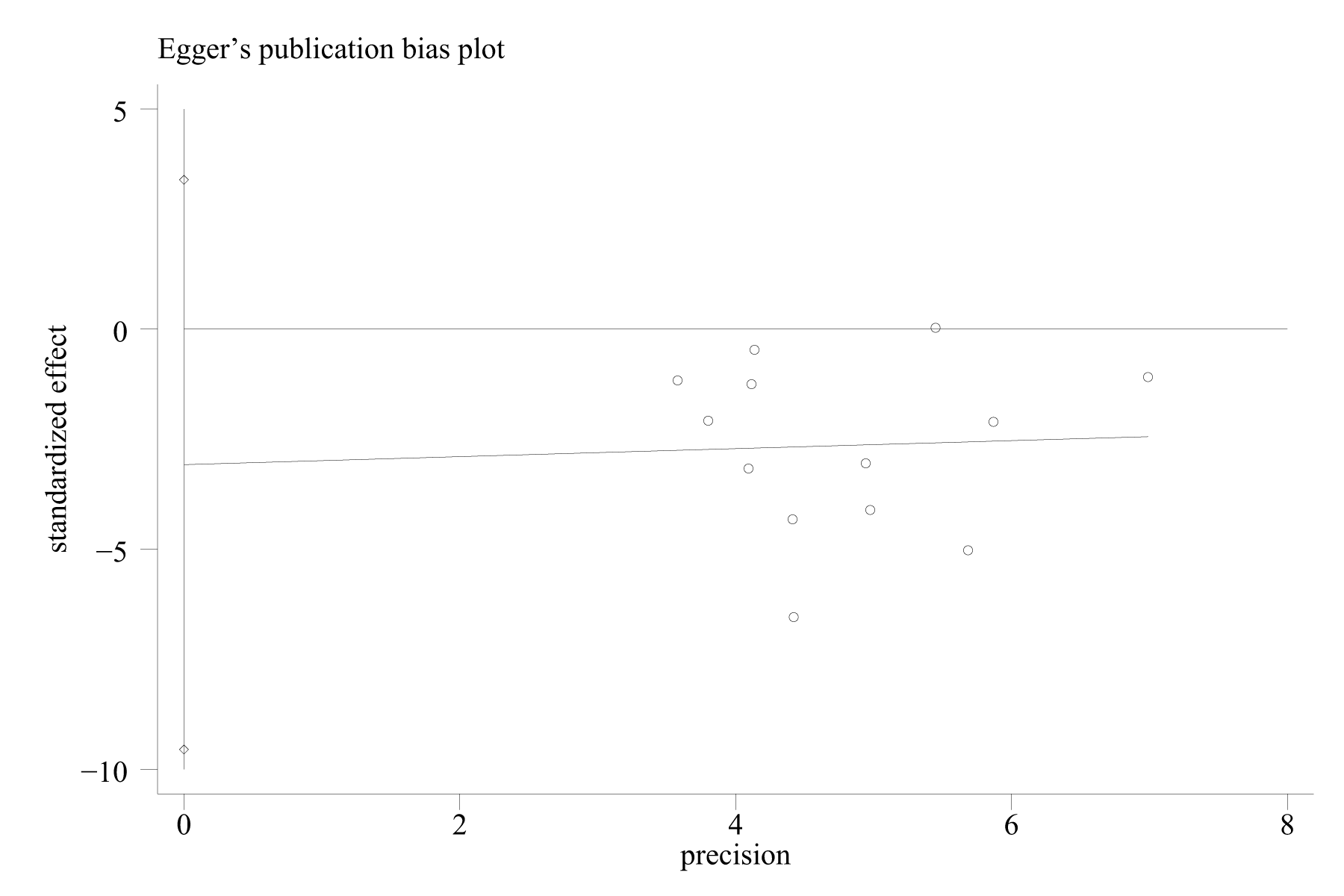


**Supplementary Figure 17** Egger's Publication Bias Plot for INFERIOR RNFL in COPD Versus Healthy Subjects


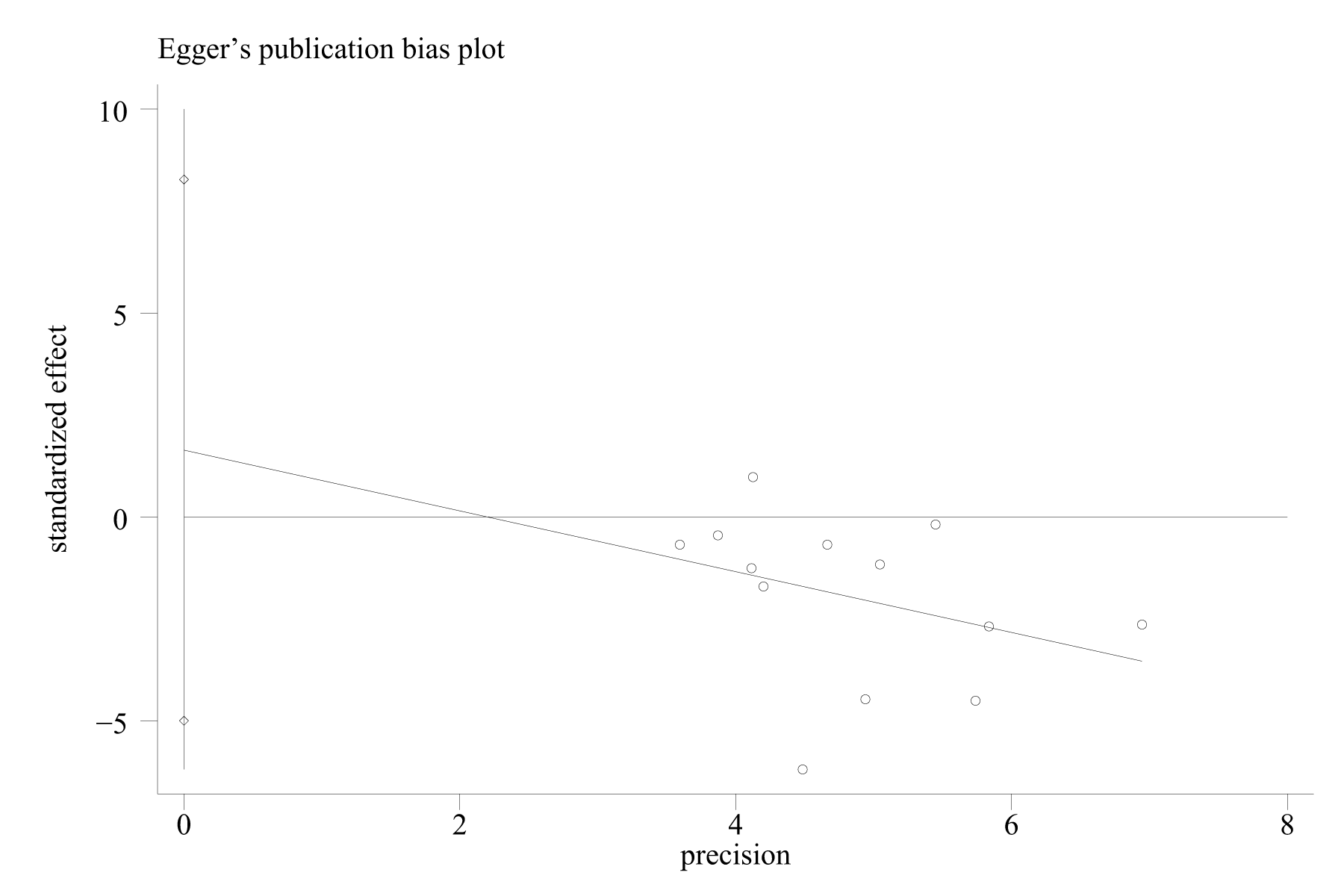


**Supplementary Figure 18** Egger's Publication Bias Plot for NASAL RNFL in COPD Versus Healthy Subjects


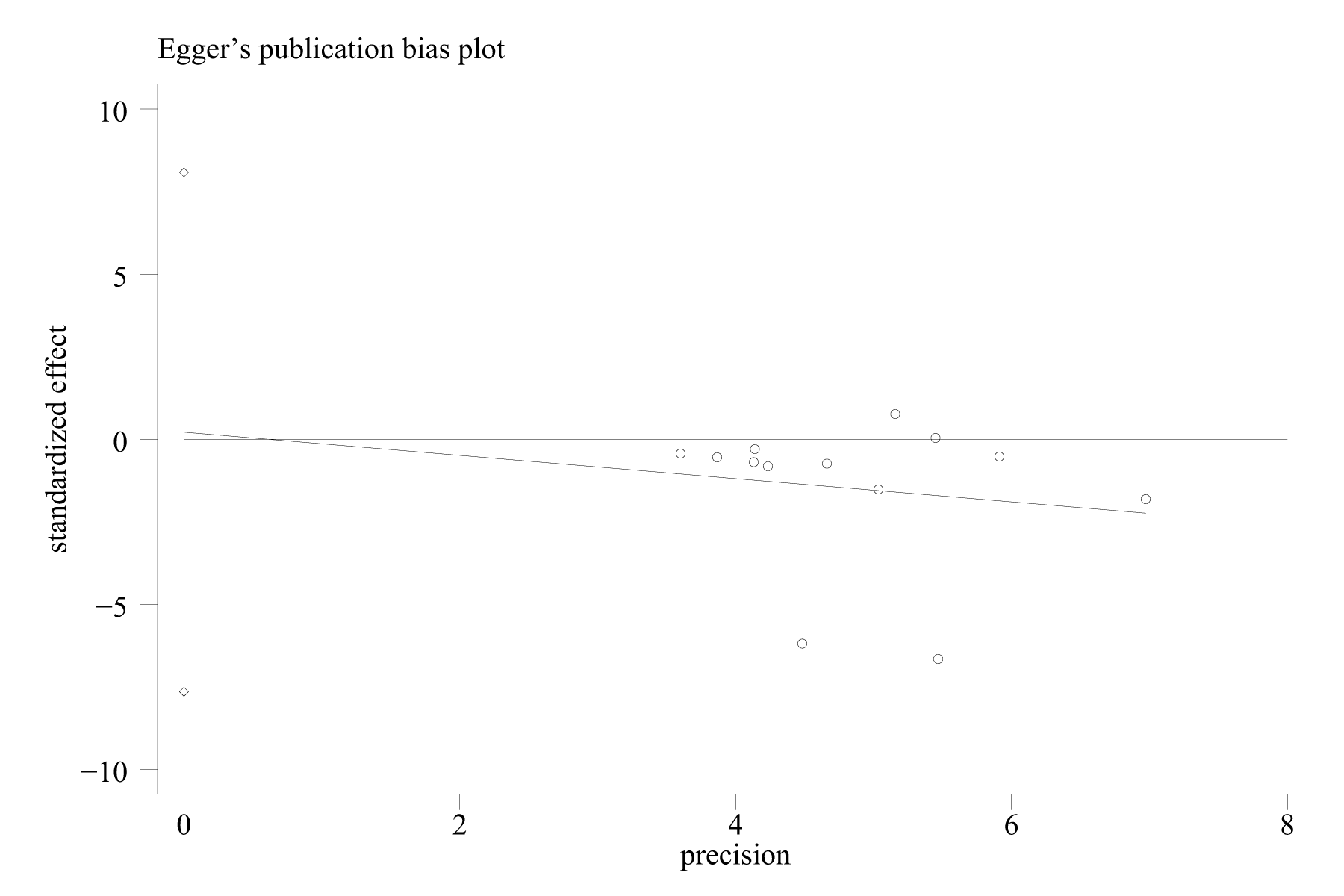


**Supplementary Figure 19** Egger's Publication Bias Plot for SUPERIOR RNFL in COPD Versus Healthy Subjects


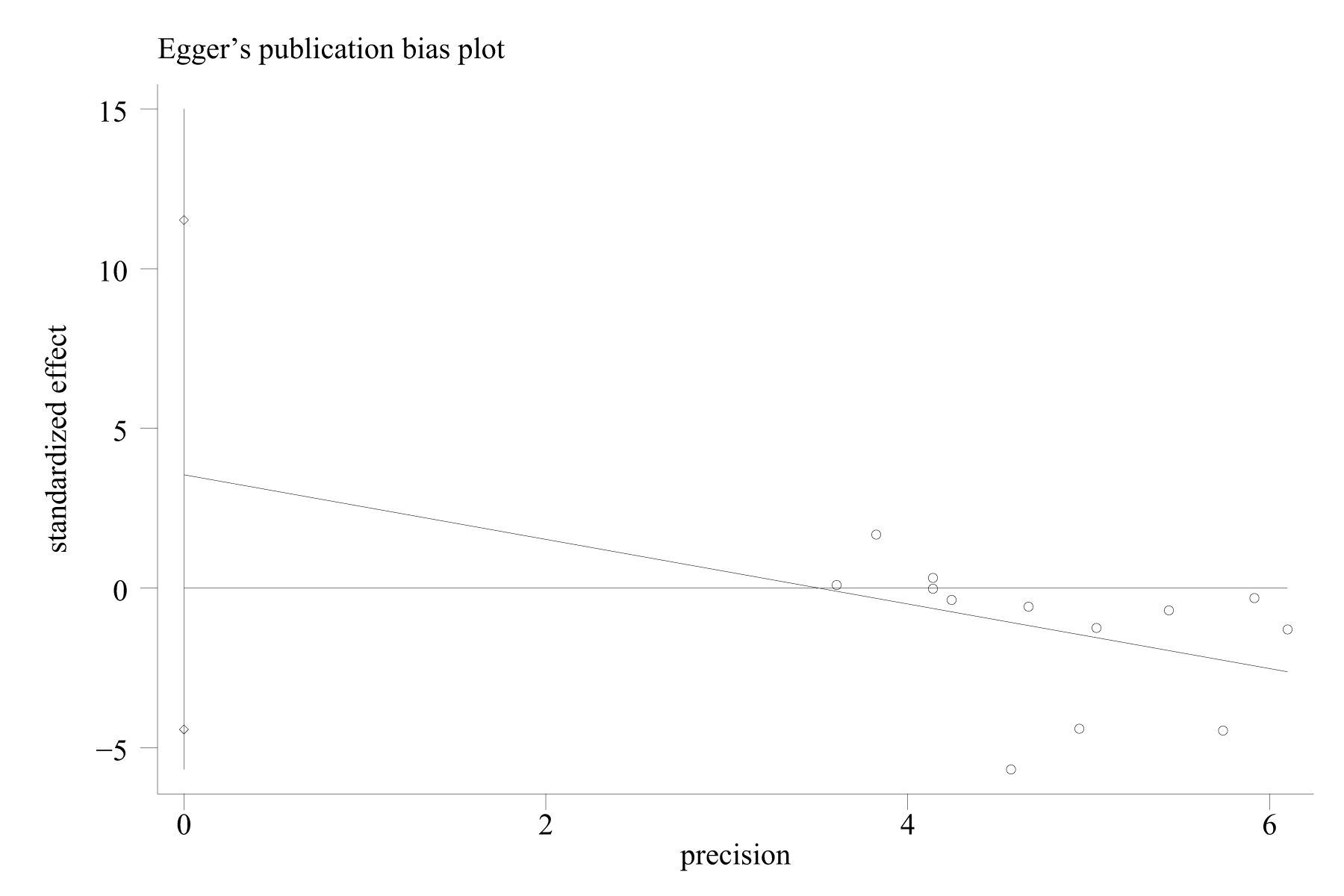


**Supplementary Figure 20** Egger's Publication Bias Plot for TEMPORAL RNFL in COPD Versus Healthy Subjects
